# Extent, intensity and drivers of mammal defaunation: a continental-scale analysis across the Neotropics

**DOI:** 10.1038/s41598-020-72010-w

**Published:** 2020-09-15

**Authors:** Juliano A. Bogoni, Carlos A. Peres, Katia M. P. M. B. Ferraz

**Affiliations:** 1 School of Environmental Sciences, University of East Anglia, Norwich, NR4 7TJ UK; 2 Laboratório de Ecologia, Departamento de Ciências Florestais, Manejo e Conservação de Fauna (LEMaC), Escola Superior de Agricultura “Luiz de Queiroz”, Universidade de São Paulo, Piracicaba, SP 13418-900 Brazil; 3 Departamento de Sistemática e Ecologia, Universidade Federal da Paraíba, João Pessoa, Paraíba 58051-900 Brazil

**Keywords:** Biodiversity, Community ecology, Conservation biology

## Abstract

Neotropical mammal diversity is currently threatened by several chronic human-induced pressures. We compiled 1,029 contemporary mammal assemblages surveyed across the Neotropics to quantify the continental-scale extent and intensity of defaunation and understand their determinants based on environmental covariates. We calculated a local defaunation index for all assemblages—adjusted by a false-absence ratio—which was examined using structural equation models. We propose a hunting index based on socioenvironmental co-variables that either intensify or inhibit hunting, which we used as an additional predictor of defaunation. Mammal defaunation intensity across the Neotropics on average erased 56.5% of the local source fauna, with ungulates comprising the most ubiquitous losses. The extent of defaunation is widespread, but more incipient in hitherto relatively intact major biomes that are rapidly succumbing to encroaching deforestation frontiers. Assemblage-wide mammal body mass distribution was greatly reduced from a historical 95th-percentile of ~ 14 kg to only ~ 4 kg in modern assemblages. Defaunation and depletion of large-bodied species were primarily driven by hunting pressure and remaining habitat area. Our findings can inform guidelines to design transnational conservation policies to safeguard native vertebrates, and ensure that the “empty ecosystem” syndrome will be deterred from reaching much of the New World tropics.

## Introduction

The Neotropical realm, spanning from 30.5° N to 59.5° S, hosts the most species-rich mammal fauna on Earth^1,2^. This realm contains 155 ecoregions containing a wide range of habitat types and distinct biotas, reflecting a bewildering continental-scale array of fauna and flora^3–5^, leading to the highest terrestrial and freshwater biotic diversity, including over 1,600 mammal species. However, since the European conquest over five centuries ago, Neotropical biodiversity has been gradually depleted in many major ecoregions (e.g.,^6–8^). Several chronic anthropogenic drivers, including habitat loss, overhunting, intentional or accidental wildfires, diseases and the introduction of alien species^9,10^ increasingly threaten the Neotropical mammal fauna. A large body of evidence to date reveals the overwhelming decline of local to regional scale Neotropical mammal diversity, except for some of the most remote areas far removed from human settlements such as large tracts of roadless Amazonian forests^11^.

Modern drivers of local biotic depletion accelerated since the 1970s across the tropics. Chief anthropogenic disturbances that lead to declines, local extinctions, and cascade-effects of vertebrate species losses include access to previously remote forested areas via new roads^12^, expansion of agribusiness frontiers^13^, wildfires fueled by climate change^14^, mounting hunting pressure^15^, relaxation of environmental law enforcement^16^, and synergistic combinations between these and socioeconomic stressors that limits access to dietary protein.

Since the European conquest, defaunation either in terms of population depletion or complete local extirpation can result from several chronic to cumulative processes including habitat loss and fragmentation, overharvesting, and disease outbreaks^17^. A generalized faunal depletion of otherwise undisturbed natural ecosystems is typically induced by unsustainable hunting^18,19^. Otherwise suitable but “empty” habitats are expected to lead to marked failures in ecosystem functioning^9,20^. Mammals play critical roles in both natural and anthropogenic ecosystems, ranging from stabilizing plant demographic dynamics to top-down control of prey populations to supporting detritivore food webs^21–23^. These ecological roles are strongly linked to multiple levels of diversity, which are highly variable across the Neotropical realm. Undisturbed equatorial rainforests may contain assemblages of up to 180 sympatric mammal species—more than 20% of which may consist of medium- to large-bodied species^24,25^—while diversity gradually decays at higher latitudes and elevations.

Yet historical simplification, dwindling population sizes and range shrinkages of mammal faunas worldwide are undermining their ecological functions^20,26^. Despite mounting but patchy empirical evidence of mammal population and assemblage declines, no study to date has documented the intensity and spatial extent of mammal defaunation across an entire tropical realm based on local occurrence data. Here, we present observed data from more than a thousand contemporary mammal assemblages distributed across all Neotropical provinces to show the magnitude, spatial extent, and drivers of mammal defaunation throughout Meso and South America. We also compiled data on several socio-environmental variables to understand the determinants of the local defaunation footprint.

We hypothesize that defaunation is a phenomenon that may be widespread but varies in extent across the Neotropics by interspersing foci of high defaunation around heavily settled human-modified landscapes with less disturbed areas retaining some core primary habitat that has somehow remained more faunally intact. However, mammal diversity in those remaining largely intact areas should be further eroded according to regional scale gradients of anthropogenic disturbance. We also expect that high levels of defaunation (> 40%) and assemblage-wide shifts in body size should be positively related to indicators of human-induced drivers, such as (i) the human footprint index and (ii) fire frequency and intensity, and (iii) local hunting pressure being influenced by habitat type and protected areas. On the other hand, defaunation and changes in the size structure of mammal assemblages should be negatively related to the proportion of remaining natural habitat. Native vegetation amount should therefore buffer the disassembly of local mammal faunas. The size structure of contemporary mammal assemblages should also be differentially affected by independent drivers, as large-bodied species may decline, for example, due to hunting pressure, but may benefit by their greater vagility and typically lower habitat specificity in highly fragmented landscapes. Local extinction responses in large-bodied species—typically artiodactylans, perissodactylans, and large carnivores—to different environmental stressors may not be straightforward, depending on the local history of human structural and non-structural disturbance. Finally, we discuss the current limitations in the body of empirical data on co-occurring mammals, with a view of enhancing this research agenda worldwide.

## Methods

### Contemporary real-world assemblages

**Figure 1:**
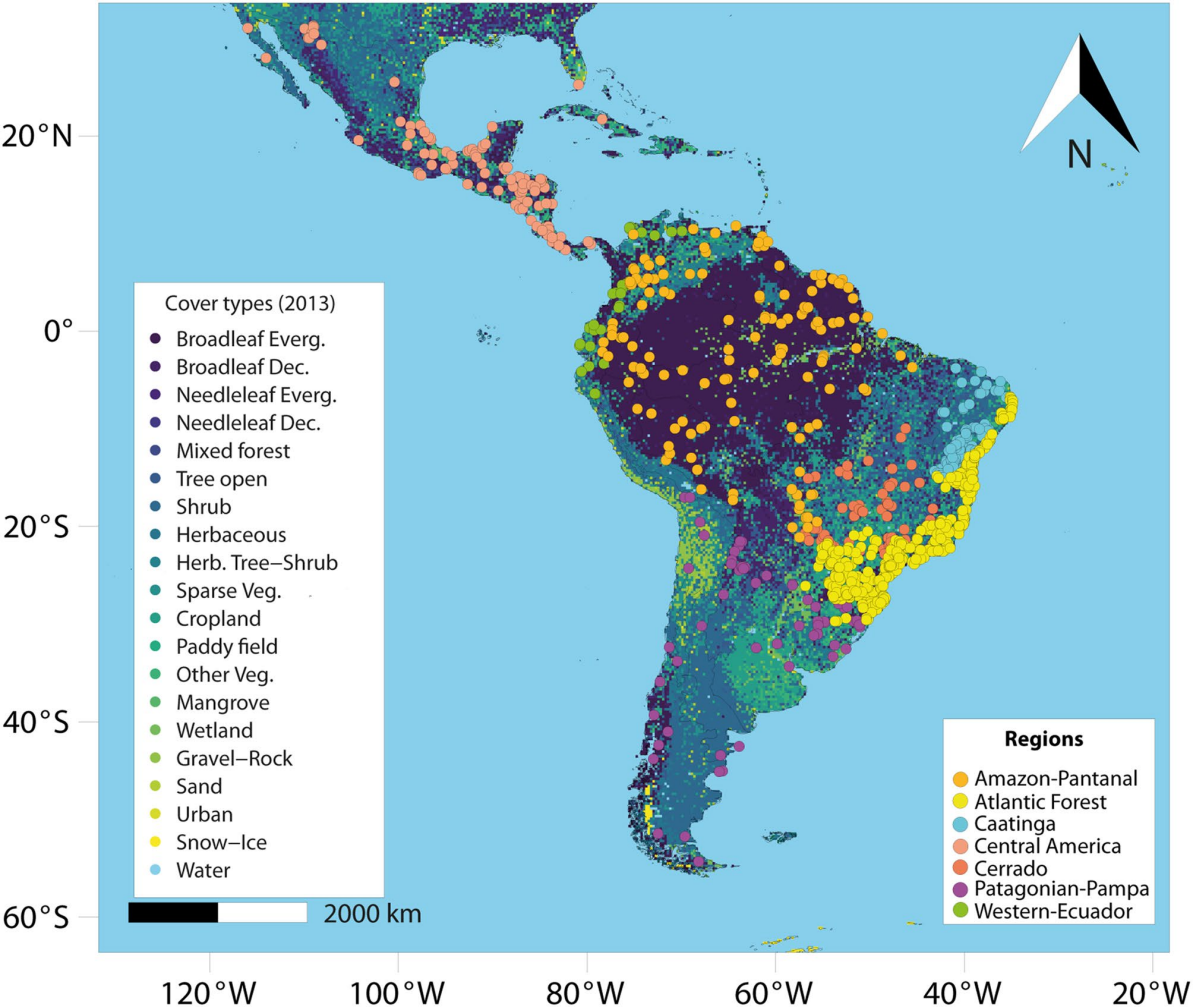
Distribution of 1,029 contemporary mammal assemblages constructed between 1983 and 2020, based on field surveys throughout the Neotropical realm. Map background and land cover are based on National Mapping Organizations (GLCNMO, MODIS data from 2013, Version 3) according to Kobayashi et al.^40^. The map was generated using R 3.5.3^52^ (https://www.r-project.org/).

From 2015 to 2020, we carried out a comprehensive survey of the literature reporting known modern assemblages of medium- to large-bodied mammals (adult body mass ≥ 1 kg) across the Neotropical realm that was either published or *in press* by early 2020. To do so, we scrutinized search engines (e.g., Scopus, Web of Science), indexing relevant sites (e.g., Google, Google Scholar), relevant Latin-American journals (e.g., Therya, Mastozoología Neotropical), recent data papers (i.e.,^27,28^), and key books containing information on well-studied Neotropical sites (e.g.,^29–31^). We controlled for redundancy in study sites using the combination of main author surname, publication year, and georeferenced coordinates. This extensive literature search was based on the combination of several keywords in four languages (English, Spanish, Portuguese and French) as follows: “mammals (or all major orders)” AND “distribution (or richness, or inventory/survey)” AND/OR “assemblage(s) (or community(ies) or richness)” AND/OR “Amazon forest (or other biomes, states or countries). Based on this approach, we were able to compile observed data on 1,029 medium- to large-bodied mammal assemblages, distributed across 23 countries from Mexico to Argentina (Fig. 1; Supporting Information S1). This database is comprised of inventories based on camera-trapping (28.5% of all sites), interviews (19.7%), active searches and line-transect censuses (16.1%), and a combination of two or more sampling methods (35.8%).

Some inconspicuous species or congeners with recent taxonomic revisions listed in the inventories (i.e., *Coendou* spp., *Bradypus* spp., *Sphiggurus* spp., and *Leopardus tigrinus*/*guttulus*) and medium- to large-bodied primates (i.e., *Alouatta* spp., *Aotus* spp., *Ateles* spp., *Brachyteles* spp., *Cacajao* spp., *Chiropotes* spp., and *Sapajus*/*Cebus* spp.) were grouped at the genus level. We further retained some small-bodied (≌ 500–1,000 g) species of Dasyproctidae (*Myoprocta* spp.), Leporidae (*Sylvilagus* spp.), and Mustelidae (*Spilogale* spp. and *Lyncodon* spp.) amounting to a total of mammal 165 (eco) species. Species smaller than 500 g (e.g., many rodents and marsupials) were removed from the analysis to minimize overinflating false-negatives and/or misidentifications.

### Presumed historical assemblages

To define the historical presence of any given species across all 1,029 sites, we first obtained the full putative assemblage assuming likely occurrences based on known geographic range polygons obtained from the IUCN (2016)^32,33^. We then combined the presumed presence/absence matrix with the observed matrix to correct for any false-absences derived from IUCN range polygons. We acknowledge that IUCN range polygons are built based on partially available information, rather than true local occurrence or distributional data, thereby potentially overestimating the number of co-occurring species at any given site. However, data on mammal occurrence derived from IUCN range polygons are widely used in conservation ecology studies (e.g.,^32,34^), comprise the only available dataset with which putative assemblages can be constructed at continental to global scales (e.g.,^35^), and are appropriate for well-studied taxa such as mammals^36^. Data on species body size and home range size were sourced from Jones et al.^37^ and Wilman et al.^38^.

### Environmental co-variables

Given the information available in each site-specific inventory, we obtained the following co-variables: (1) georeferenced coordinates (decimal degree); (2) site denomination (e.g., national park, private reserve, indigenous territory, private landholding); and (3) protected area (yes/no). Based on the geographic coordinates of each inventory locality, expressed as a latlong projection (Datum WGS 84), we further obtained several landscape variables at 4.5-km radial buffer (which represents the mean species home range size sourced from Wilman et al.^38^). Covariates extracted and then averaged over the buffer area included the (1) human footprint index (HFI) v.2 (30-arc-sec resolution^39^); (2) land cover v.3/2013 at 15-arc-sec resolution (Fig. 1)^40^ providing the remaining of habitat area (HA, %) by coalescing all natural habitat land-cover (e.g., native forest, native grasslands, sparse shrublands); and (3) a combination of fire intensity and frequency, by weighing the average burn intensity by the frequency of monthly events (0.1 degree resolution, dated from the second semester of 2000, 2008–2009, and 2018–2019^41^. HFI and land cover data covered time periods of 1993–2009 and 2003–2013, respectively. The multitemporal fire data match the timing of 97.6% of all assemblages surveyed, which on average were published 3.6 years after each in situ inventory had been conducted^42^. The potential predictors we used therefore provide a good representation of simultaneous environmental stressors exerted upon the mammal assemblages compiled, 89.1% (917) of which were surveyed between 2009 and 2019.

We further obtained the province and ecoregion of each site based on polygons sourced from Morrone^43^ and Olson et al.^4^. We segmented the data into seven major biogeographic regions as following: (1) Amazon-Pantanal (provinces: Guianan, Imerí, Madeira, Napo, Pantepui, Pará, Paramo, Rondônia, Roraima, Sabana, Trinidad, Ucayali, Venezuelan, and Xingu-Tapajós); (2) Atlantic Forest (Atlantic, Araucaria, and Parana); (3) Caatinga (Caatinga); (4) Central America (Bahama, Balsas, Chiapas, Cuban, Florida peninsula, Guatuso-Talamanca, Hispaniola, Mosquito, Pacific-Lowlands, Puntarenas, Sierra-Madre, Sonora, Transmexican-Volcanic, Veracruzan, and Yucatán); (5) Cerrado (Cerrado); (6) Patagonian-Pampa (Atacaman, Chacoan, Desert, Monte, Pampean, Patagonian, Prepuna, Puna, and Yungas); (7) Western-Ecuador: (Cauca, Chocó-Darién, Ecuadorian, Guajira, Magdalena, and Western Ecuador). We also used this segmentation to cross-validate our data layer extraction. In doing so, we assessed the spatial configuration of the extreme-range of local defaunation for each ecoregion within Google Earth^44^ imagery. Moreover, for each site, we classified the habitat type (HT) as either predominantly open areas (e.g., savannas, grasslands, shrublands) or predominantly forest areas (e.g., broadleaf, coniferous) based on the ecoregion characteristics sourced from Olson et al.^4^.

### Hunting pressure index

We generated a hunting pressure index (HPI) for each site based on a set of factors that can either inhibit or intensify hunting pressure (Table 1; Supporting Information S2). We first extracted data from spatial layers (latlong projection, datum WGS 84), based on a 4.5-km radial buffer around the geographic coordinates of each site, on the following features that inhibit hunting pressure: (i) elevation (site scale), (ii) artificial lights, (iii) protection status, and (iv) the ratio between net primary productivity (NPP) and a proxy of native vegetation biomass (by multiplying the proportion of habitat area (HA) by the mean vegetation height). Conversely, we extracted data for variables that likely intensify hunting pressure: (i) NPP, (ii) the 95th percentile of the species body size distribution in historical assemblages (site), (iii) a spatial metric of human disturbance (HFI), (iv) absolute latitude (site), (v) purchase power (dis)parity (PPP), and (vi) water bodies (Supporting Information S3). We summarized the reasons for using each of these variables in Table 1 (see also Supporting Information S2). Using this dataset, we obtained a value describing the degree to which hunting pressure was inhibited (Eq. 1), derived from prior equations based on observed values at *i*_th_ assemblage as a function of the maximum value of any assemblage (from *i*_th_ to *j*_th_ assemblage) (Table 1). Conversely, we used the same approach to obtain a value of hunting severity (Eq. 2). Given these two values, we derived the HPI metric (Eq. 3) by subtracting Eq. 1 (inhibition) from Eq. 2 (intensification) (see Table 1; Supporting Information S4). Thus, our HPI metric can range from − 1.0 (low pressure) to + 1.0 (high pressure). To formally assess the HPI metric, we applied the Bayes theorem^45^ (Supporting Information S5) to all values derived from Eq. 1 versus Eq. 2, expecting a significant linear relationship between the Bayes-posterior probability and our proposed HPI metric. The Bayes theorem—given any two events A and B and the conditional probability of A given B—is denoted by P(A|B)^45^. Therefore, the hunting intensification (P(Eq. 2)) is conditioned on the factors that inhibit hunting (P(Eq. 1)). The posterior probability value derived from the Bayes theorem (i.e., P(Eq. 2|Eq. 1) is more strongly related to the factors that intensify, rather than the factors that reduce hunting pressure. The subtraction value of “intensification–inhibition” (i.e., intensification minus inhibition or HPI) is correlated with the probability value derived from the Bayes theorem, whereby hunting intensification (Eq. 2) given the factors that inhibit hunting (Eq. 1) are true (see more details in the Supporting Information S5). Using this approach, we found a significant linear relationship [R^2^_adj_ = 0.69; *p* < 0.001] between HPI and the posterior probability of hunting intensification given the hunting inhibition values (Eq. 2|Eq. 1). This indicates that hunting pressure increases as a function of greater intensification, although the HPI value depends on both sets of metrics (Supporting Information S5). Given that we identified a spatial autocorrelation in HPI [M_obs_ = 0.13; M_exp_ =  − 0.01; *p* < 0.001], we used a kriging interpolation approach^46^ to derive a georeferenced map of HPI (≌ 4.5-km pixel resolution) pruned to fit the Neotropical realm boundaries of Morrone^43^ (Supporting Information S6). Following the same interpolation approach, we also mapped the Bayes-posterior probability to all values derived from Eq. 1 versus Eq. 2 to provide a spatial overview of the relationship between HPI and the Bayes-posterior probability values (Supporting Information S7).

**Table 1:** Variables and their justifications used to derive the hunting pressure index (HPI) at 1,029 sites throughout the Neotropical realm. Source references, scales and equations used are also included.

Variables	Var. type	Justification	Refs	Scale	Prior equations
**Hunting inhibition**					
Elevation	c	High-elevation decreases species richness and abundance, and renders hunting sites less accessible to hunters	^1,2^	Site	$e_{i} = e_{i} {/}max\left( {e_{i,j} } \right)$
Artificial lights	c	Nocturnal lights inhibit hunting, and represents a strong proxy of purchase power in acquiring alternative animal protein	^3^	4.5-km	$l_{i} = l_{i} {/}max(l_{i,j} )$
Protected area^a^	b	Law enforcement and jurisdiction of wildlife protection	^4,5^	Site	$PA_{i} = 1(yes) \;or\; 0(no)$
NPP/Plantbiomass ratio	c	Presumably areas with high NPP and low native vegetation cover have higher availability of domestic livestock protein	–	4.5-km	$NB_{i} = NPP_{i} {/}(vh_{i} . nc_{i} ){\text{/max}}(NPP_{i,j} {/(}vh_{i,j} . nc_{i} ,j{))}$)
**(Eq. 1) Inhibition value**^3^					$AV_{i} = \overline{x} \left( {e_{i} , l_{i} , PA_{i} , NB_{i} } \right)$
**Hunting intensification**					
NPP	c	NPP leads to increases in prey abundance	^7^	4.5-km	$NPP_{i} = NPP_{i} {/}max(NPP_{i,j} )$
Assemblage-wide body mass distribution	c	Hunters operate under the tenets of optimal foraging theory	^9^	–	$BS_{i}^{95th} = BS_{i}^{95th} {\text{/max(}}BS_{i,j}^{95th} {)}$
HFI	c	Hunter access via roads and other infrastructure	^10^	4.5-km	$HFI_{i} = HFI_{i} {/}max(HFI_{i,j} )$
Absolute latitude	c	Species richness decreases away from the equator	^11^	Site	$abs(L_{i} ) = 1 - abs(L_{i} ){\text{/max}}(abs(L_{i,j} ))$
Purchase power (dis)parity (PPP)	c	Motivation to hunt is higher if alternative animal protein is unaffordable	–	4.5-km	$PPP_{i} = 1 - PPP_{i} {/}max(NPP_{i,j} )$
Water bodies	c	Hunter access by water	^12^	4.5-km	$wb_{i} = wb_{i} {/}max(wb_{i,j} )$
**(Eq. 2) Intensification value**^3^					$PV_{i} = \overline{x} \left( {NPP_{i} , BS_{i}^{95th} , HFI_{i} , abs(L_{i} ), PPP_{i} ,wb_{i} } \right)$
**(Eq. 3) Hunting pressure index (HPI)**					$HPI_{i} = PV_{i} - AV_{i}$

Codes and acronyms: 1: Lomolino^111^; 2: Bogoni et al.^50^; 3: Gaynor et al.^112^; 4: Joppa et al.^72^; 5: Gray et al.^73^; 7: Waide et al.^113^; 8: Oliveira and Begossi^114^; 10: Benítez-López et al.^78^; 11: Cardillo^115^; 12: Antunes et al.^7^; c, continuous; b, binary; NPP, net primary productivity; HFI, human footprint index.

^a^The presence of protected area contributes 25% to of the mean hunting inhibition.

### Data analysis

#### Defaunation index

We calculated a site-specific naïve defaunation index (DI) based on the ratio between the observed (contemporary) and the expected (historical) species richness. DI therefore ranges from 0.0 (faunally intact) to 1.0 (completely defaunated). Some species are naturally rare for several reasons^47,48^, leading to an increase in the probability of false-absences which can only be detected by large sampling efforts^49^, thereby potentially overinflating DI^50^. We therefore controlled for this issue by conservatively adjusting the naïve DI. We based this adjusted defaunation index (DI_adj_) on a confusion matrix approach^51^ based on confronting the contingency presence-absence matrices in both the observed and presumed historical assemblages. This approach estimates rates of presumed false-absences in contemporary assemblages by calculating the cross-tabulation of observed and predicted classes (e.g., contemporary vs. historical presences given the 2 × 2 table that also contains absences). This approach thus assumes historical presences as the baseline from which rates of potential false-absences were derived in light of contemporary absences and relict presences^51^. For example, if a historical assemblage retains a species richness (presences) of 25 species (i.e., 140 real absences) that given a modern inventory has been reduced to 11 species (i.e., 140 real absences + 14 potential absences [i.e. 25–11]), the confusion matrix can derive predictive values of how many modern absences can be defined as false-negatives given the sensitivity, specificity and prevalence deriving the positive predictive values (PPV)^51^. In this working example, these values are sensitivity = 140/154 (i.e., = 0.91); specificity = 11/(25 + 11) (i.e., = 0.31); prevalence = (140 + 154)/(140 + 154 + 25 + 11) (i.e., = 0.89); and PPV = (0.91_[sensitivity]_ × 0.89 _[prevalence]_)/((0.91_[sensitivity]_ × 0.89 _[prevalence]_) + (1 − 0.31_[specificity]_ × (1 − 0.89_[prevalence]_)) (i.e., PPV = 0.91). We therefore corrected the naïve defaunation estimates using the false-absence ratio (1 − PPV) for each site, which we also derived from other statistics (e.g., accuracy, accuracy-CI, and McNemar *p* value)^51^. This analysis was performed in R 3.5.3^52^ using the *caret* package^51^. We also used a kriging interpolation approach^46^ to derive a map of potential false-absence ratios across the Neotropical realm boundaries to illustrate the bias-corrected defaunation (Supporting Information S7).

#### Geographic extent and determinants of defaunation

We first examined the spatial autocorrelation of our measures of both naïve (DI) and adjusted defaunation (DI_adj._) using the Moran index^53^. Given that spatial autocorrelation was detected [M_obs_ = 0.23; M_exp_ = – 0.01; *p* < 0.001; M_obs_ = 0.21; M_exp_ = – 0.01; *p* < 0.001; respectively], we explored the site-scale spatial patterns of both defaunation indices and used a kriging interpolation^46^ to represent unsampled areas, generating a continental-wide map of DI and DI_adj_ at ≌ 4.5-km pixel resolution. We also explored the interpolation of naïve defaunation segmented for each mammalian order. Further, we used the 95th-percentile of mammal body size within any pairwise (contemporary vs. historical) assemblage to quantify compositional shifts in size structure across the entire Neotropical realm.

To disentangle the likely cause-effect relationships between potential drivers of both adjusted defaunation and assemblage downsizing, we used path analysis (i.e., structural equation modeling [SEM])^54^. SEM was implemented to simultaneously predict adjusted defaunation and downsizing as an additive function of (i) HPI; (ii) HFI; (iii) fire; (iv) habitat area (HA); (v) habitat type (HT); and protected status (PA). Results are shown using the SEM standardized coefficient, and we obtained R^2^-values based on the proportion of variance explained by each predictor^55,56^. Positive asymmetric data (i.e., HFI and fire) were previously log-transformed (log_10_ x + 1). Data analysis was performed using R 3.5.3^52^ based on the *sem* function in the *lavaan* package^57^. For the best predictors identified based on SEM, we plotted both linear and smoothed functions to present their explicit effect sizes in explaining adjusted defaunation and assemblage downsizing.

## Results

### Anthropogenic hunting footprint

**Figure 2:**
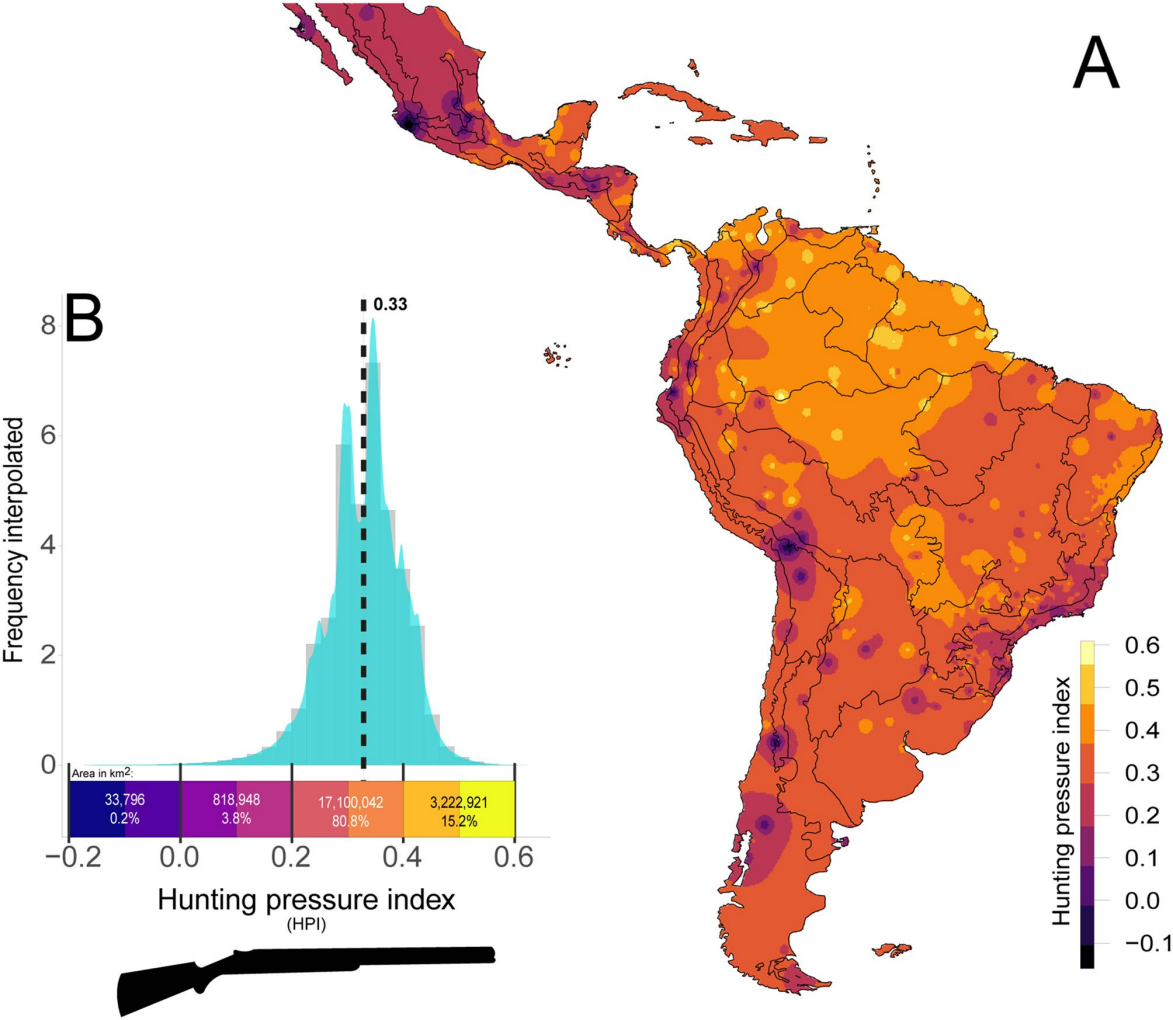
Pan-Neotropical spatial interpolation of the (**A**) Hunting Pressure Index (HPI), which in terms of total area distribution matches (**B**) the frequency distribution of degree of defaunation across this entire realm. Both the map and the histogram were equally colour-coded. Vertical dashed line indicates the mean defaunation value. The map was generated using R 3.5.3^52^ (https://www.r-project.org/).

Our hunting pressure index (HPI) revealed an overall mean value of 0.33 (± 0.14; range = – 0.17, + 0.63) for the entire Neotropical realm. Interpolated values throughout the Neotropics shows that a vast fraction of this terrestrial realm has experienced fairly high HPI values from 0.2 to 0.4, spanning a total area of ~ 17 million km^2^, which largely includes the Amazon, Cerrado, Caatinga and Patagonian regions (Fig. 2). In contrast, only 4% (~ 850,000 km^2^) of the Neotropics experienced HPI values of 0.2 or lower; these areas are mainly concentrated in naturally species-poor regions or those that had been historically most depleted (Fig. 2).

### Extent and intensity of defaunation

Considering all 1,029 putative historical mammal assemblages across the entire Neotropical realm, the mean local species richness was expected to be 31.7 (± 6.9) species reflecting 32,587 expected presences. However, contemporary assemblages on average retained only 37.5% or 11.9 (± 8.8) species per site, reflecting a total of 12,231 observed presences. This reveals an overall level of naïve defaunation across the entire Neotropics of DI = 0.63 (± 0.25). On the basis of our confusion matrix, we identified an average ratio of false-negatives in contemporary assemblages of 10.5% (± 4.0%; range = 0.0–19.2%) providing an average accuracy value of 89.9% (± 3.9%; IC_average_ = 85.0–93.7%) with only 5 sites showing a McNemar *p* value > 0.05. Adjusting the unqualified level of naïve defaunation based on the previously calculated false-negative ratio for each site, our results reveal a mean adjusted defaunation of 56.5% [DI_adj._ = 0.56 (± 0.22)]. Among the most defaunated subregions were the Caatinga of northeastern Brazil (DI_adj_ = 0.75; ± 0.14; N = 124), the Atlantic Forest domain (DI_adj._ = 0.62; ± 0.19; N = 482), and the Cerrado wooded scrublands (DI_adj._ = 0.50; ± 0.17; N = 74), in which artiodactylans locally were the most impacted mammal order (DI = 0.67; ± 0.32).

**Figure 3:**
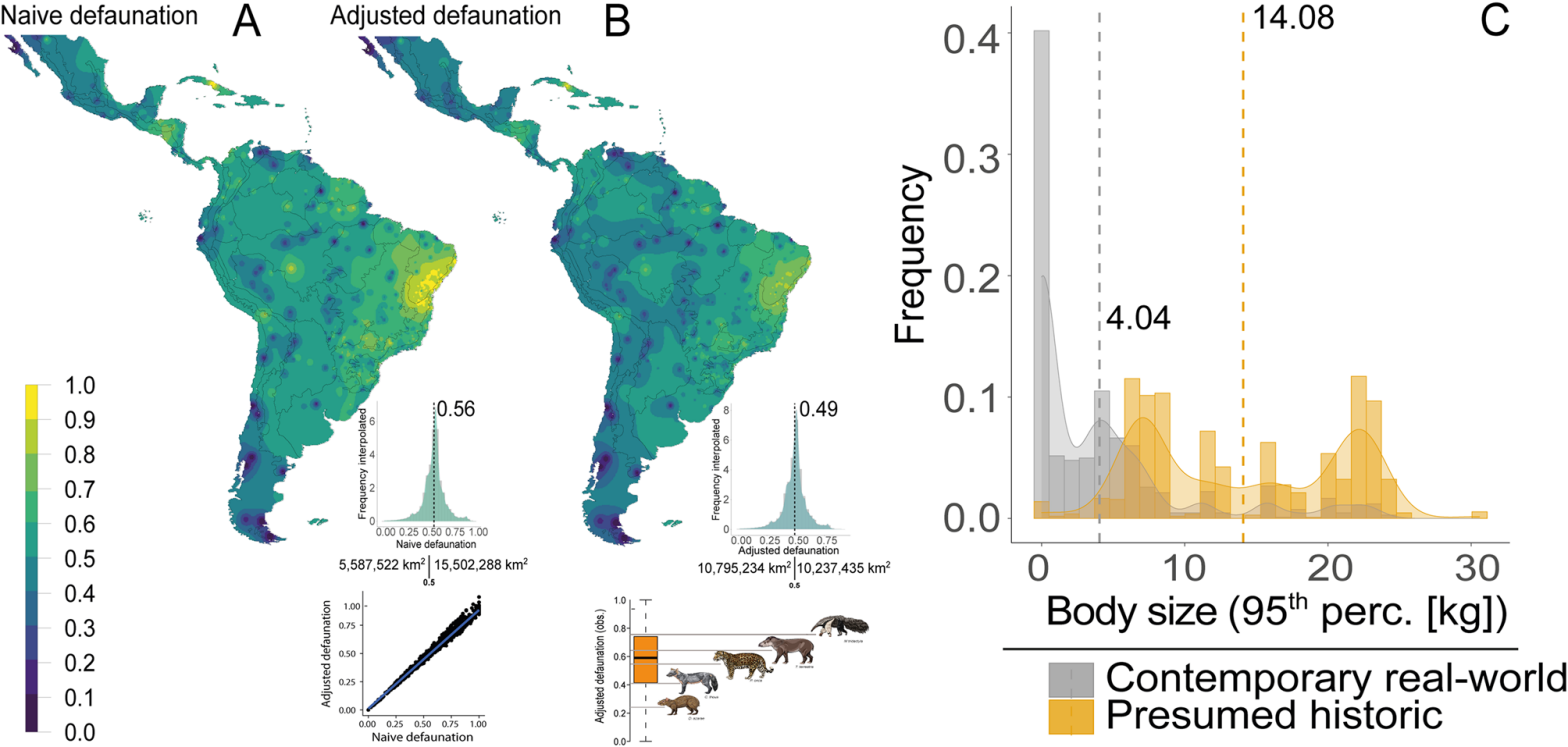
(**A**) Overall distribution of pan-Neotropical naïve defaunation (inset shows the linear relationship between the naïve and adjusted defaunation) paired with the (**B**) Adjusted defaunation and observed adjusted defaunation and an example of ranked defaunation rates for some iconic mammal species (from bottom to top: *Dasyprocta azarae*, *Cerdocyon thous*, *Panthera onca*, *Tapirus terrestris* and *Myrmecsophaga tridactyla*); and (**C**) the distribution of assemblage-scale values of the 95th percentile of body mass in both historical and contemporary assemblages across the Neotropical realm represented by their respective distributions (vertical bars) and polynomial smoothed functions (curves). The maps were generated using R 3.5.3^52^ (https://www.r-project.org/).

Our continental-scale interpolation showed high variation in defaunation across all Neotropical provinces (Fig. 3; Supporting Information S8), with the most severe levels concentrated in Mesoamerica, the entire Caatinga biome, and the northern portion of South America. Interpolation-based adjusted defaunation lower than 25% spans a Neotropical area of 435,260 km^2^; values from 25.1 to 50% span 10,359,974 km^2^, values from 50.1 to 75% span 9,905,458 km^2^, and > 75% defaunation spans an area of 331,995 km^2^. Regions that once hosted relatively depauperate mammal faunas (> 1 kg), such as the Patagonia-Pampa biomes of South America’s southern cone, exhibited intermediate levels of defaunation (DI_adj._ = 0.38; ± 0.21; N = 63; Fig. 3; Supporting Information S8). The Pan-Amazon and the Pantanal wetlands comprised the most faunally intact regions (DI_adj._ = 0.44; ± 0.19; N = 150), but their mammal faunas are now being gradually eroded along peripheral agricultural frontiers (Fig. 3). Considering the geopolitical breakdown of defaunation, Neotropical countries showing the heaviest defaunation burden include Nicaragua (DI_adj._ = 0.63; N = 11), Honduras (DI_adj._ = 0.62; N = 18), Brazil (DI_adj._ = 0.61; N = 767), Colombia (DI_adj._ = 0.54; N = 37), and Peru (DI_adj._ = 0.43; N = 25).

**Figure 4:**
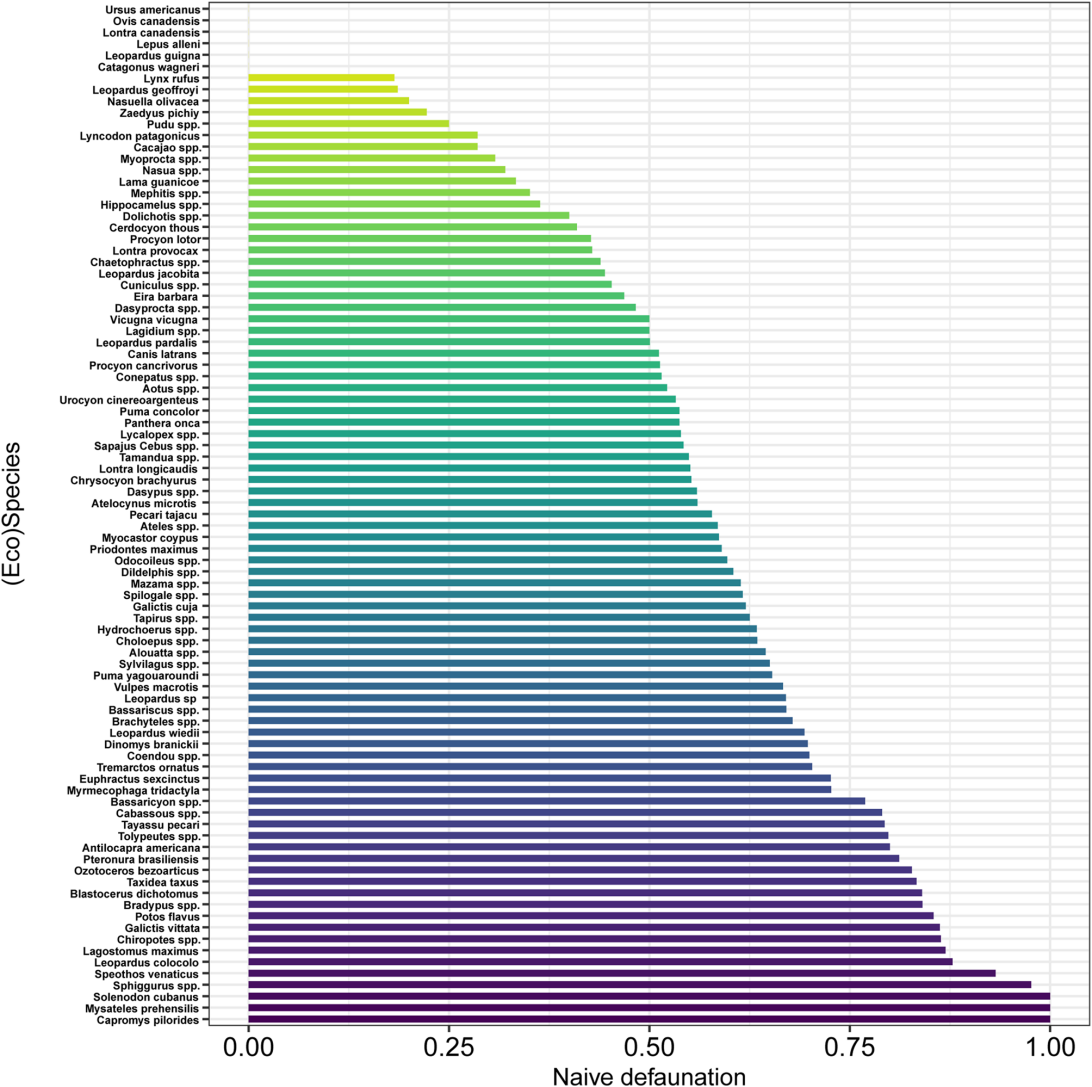
Observed rates of naïve site occurrence of medium- to large-bodied mammal (eco)species across 1,029 mammal assemblages distributed throughout the Neotropical realm. Species are listed top to bottom from the highest to the lowest occurrence rates; darker colours represent species with the lowest occurrence or highest defaunation rates.

**Figure 5:**
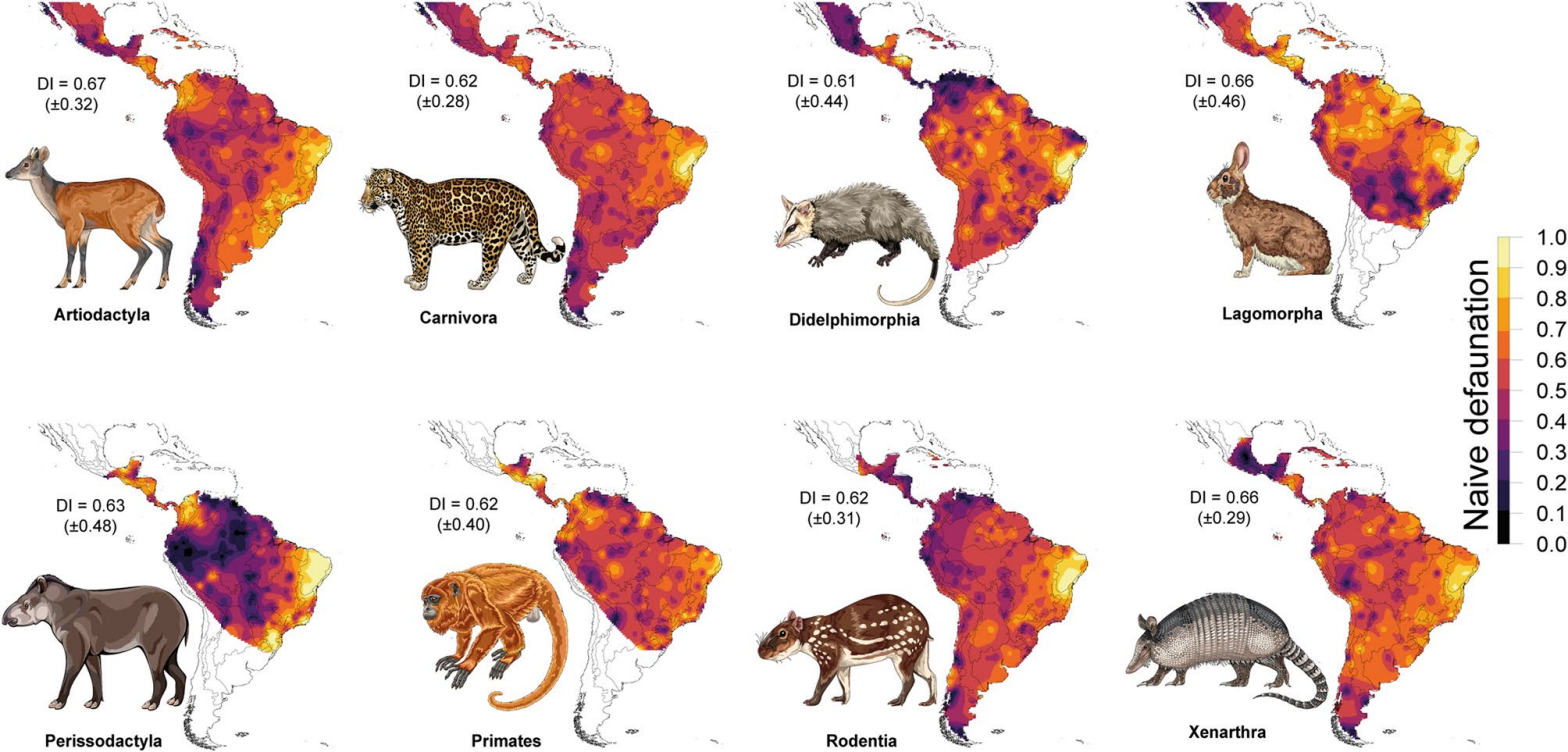
Interpolation-based geographic distribution of the naïve defaunation index (DI) broken down by mammal orders across the Neotropics. Maps were pruned by the averaged limit of the distribution of any mammal species within each order via a convex-hull approach. The order Eulipotyphla is not shown because there is only one species (*Solenodon cubanus*) in the dataset. The maps were generated using R 3.5.3^52^ (https://www.r-project.org/). Fernanda D. Abra (ViaFAUNA) kindly provided mammal species drawing used in the figure.

Assemblage-wide reductions in the size structure (i.e., downsizing) accompanied high rates of defaunation, ranging from faunally intact sites where all historical occurrences had been retained, to sites where all large-bodied species were apparently missing. The average 95th-percentile of species body mass in observed assemblages was 4.04 (± 5.6) kg, equivalent to only 28.7% of the analogous value in historical assemblages (14.08 [± 7.0] kg; see Fig. 3C). The Cerrado and Atlantic Forest biomes contained the most downsized mammal faunas (– 14.77 [± 7.1] and – 10.99 [± 5.9], respectively). In addition to artiodactylans, other highly affected orders included lagomorphs (DI_adj._ = 0.66 ± 0.46), xenarthrans (DI_adj._ = 0.66 ± 0.29), and perissodactylans (DI_adj._ = 0.63 ± 0.48) (see details in Fig. 4). Although these orders showed high geographic variation, their most defaunated sites are in the Caatinga and forests of southeastern Brazil and parts of Mesoamerica (Fig. 5).

### Environmental drivers of defaunation

**Figure 6:**
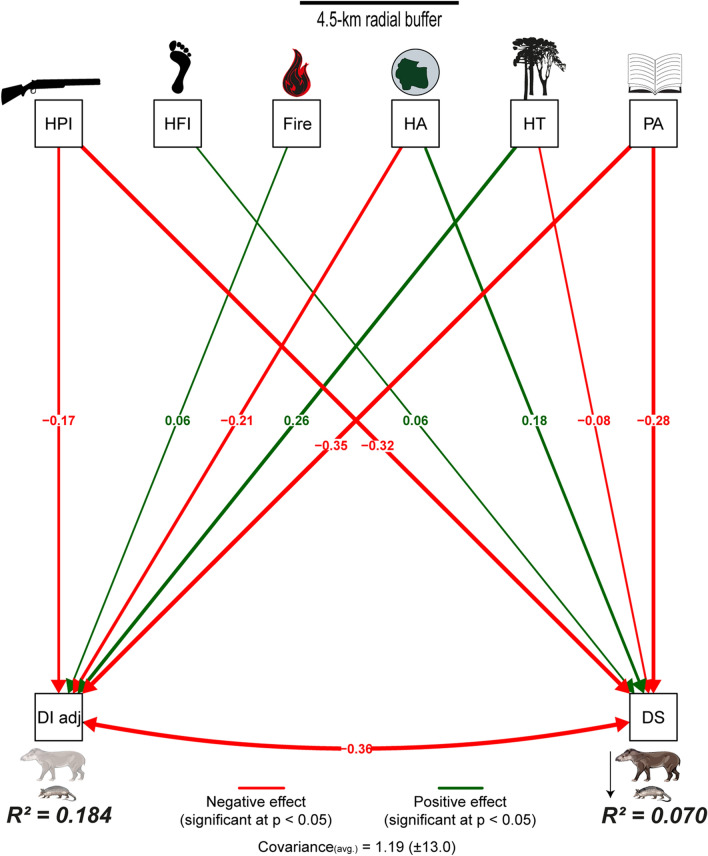
Structural equation models (SEM: path analysis) to disentangle the linear cause-effect relationships between different environmental and demographic variables and either the adjusted defaunation (left arrows) and assemblage downsizing (right arrows) across 1,029 Neotropical sites. Green and red vectors represent positive and negative effects, respectively. Thicker vectors represent stronger effects. HPI, hunting pressure index; HFI, human footprint index; HA, habitat area; HT, habitat type; PA, protected area; DI_adj._, adjusted defaunation index; and DS, assemblage downsizing. Fernanda D. Abra (ViaFAUNA) kindly provided mammal species drawing used in the figure.

**Figure 7:**
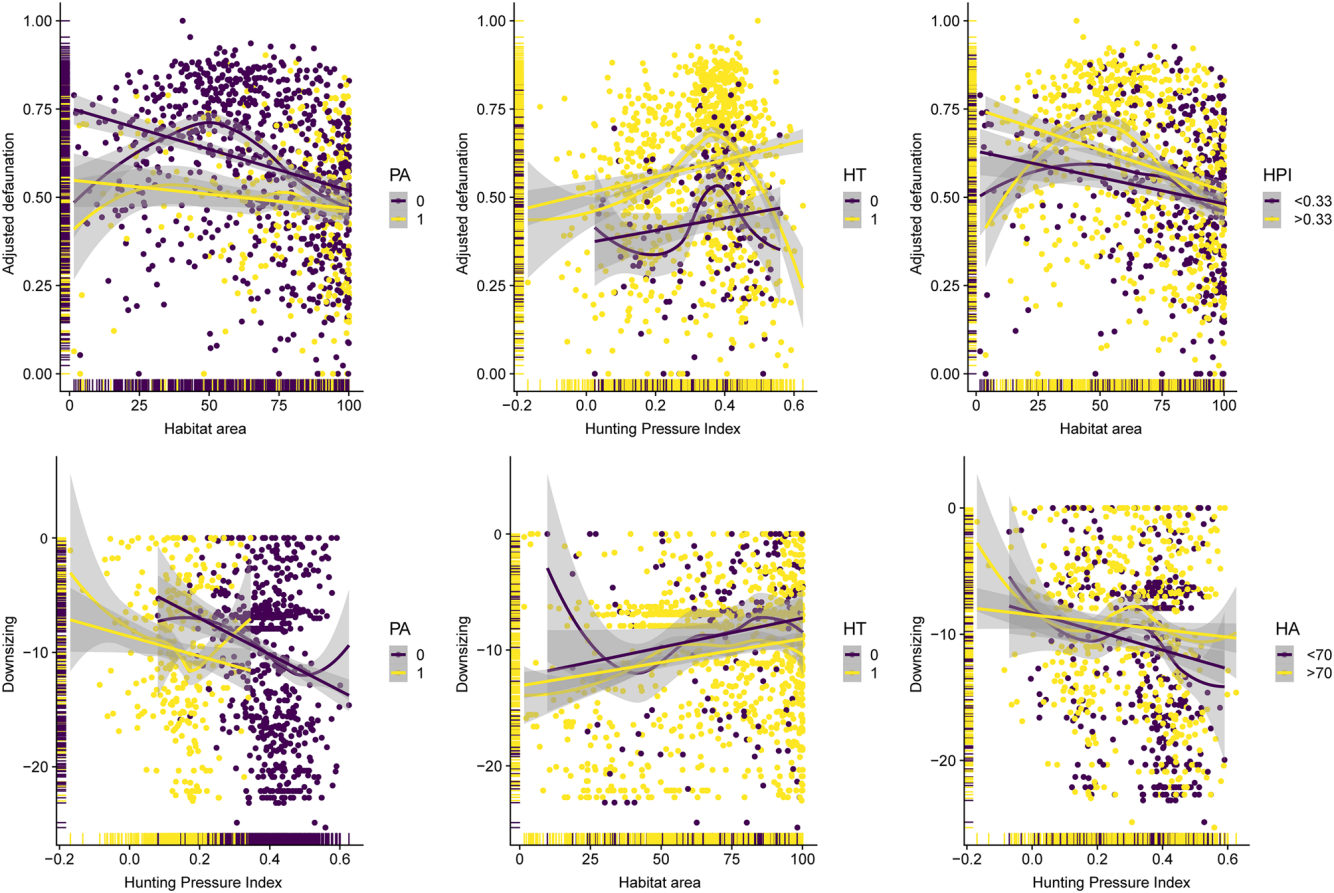
Linear and non-linear scatterplots representing the main important bivariate relationships according to the structural equation models used to predict the adjusted defaunation (top panels) and assemblage downsizing (bottom panels) across 1,029 Neotropical mammal assemblages. Purple and yellow solid circles represent different classes in categorical variables or critical thresholds in continuous variables. PA, protected area (0 = no, 1 = yes); HT, habitat type (0 = open; 1 = forest); HPI, hunting pressure index; and HA, habitat area.

Our results indicate that land protection status (PA = – 0.35; *p* < 0.001), habitat type (HT = 0.26; *p* < 0.001) and landscape-scale habitat area (HA = – 0.21; *p* < 0.001) were the most important determinants of the degree of defaunation (Fig. 6), whereby the most intact, protected landscapes retained the most intact mammal faunas, particularly in open-habitat areas. Assemblage downsizing was strongly affected by hunting pressure (HPI = – 0.32; *p* < 0.001), but this was attenuated by the effects of protection (PA = – 0.28; *p* < 0.001) and large areas of remaining habitat (HA = 0.18; *p* < 0.001; Fig. 6). Bivariate regression plots containing the main continuous (i.e., HA and HPI) and categorical variables (e.g., PA and HT), indicated that some relationships predicting both overall defaunation and assemblage downsizing were markedly nonlinear (Fig. 7).

## Discussion

Homeotherm vertebrate diversity worldwide has been persistently reduced by several leading anthropogenic drivers, including primary habitat conversion, habitat degradation and overhunting^32,58^, arguably representing the best-known component of the global biodiversity crisis. A growing corpus of evidence shows high rates of mammal population depletion, often at regional scales (e.g.,^6,8,59^), which may lead to detrimental effects on ecosystem functioning (e.g.,^9,60,61^). Here, based on the largest mammal assemblage dataset ever compiled for any major tropical realm, we show the alarming and pervasive effects of large scale human-induced extirpations. To our knowledge this is the first time this is documented at a continental-scale based on observed species occurrence data. Our results reveal the alarming extent of historical mammal assemblage decay across the Neotropics and its major ecoregions and biomes. This defaunation process can be broken down into a compositional simplification of the mammal faunas coupled with a predictable size shift in which depleted assemblages were represented by an ever smaller subset of small-bodied species. Land use change, hunting pressure, physical accessibility to wildlands and habitat degradation were the main correlates of assemblage-wide mammal extirpation against a relatively recent post-colonial background (50–500 yrs).

Severely defaunated areas reflect the highest levels of species loss for both ungulates and carnivores, representing the largest consumers and apex predators in most terrestrial ecosystems. Portions of northern South America (e.g., Colombia), Mesoamerica and large Caribbean islands, such as Cuba, Hispaniola and Jamaica, converge on similar patterns of mammal extirpation. That ungulate megaherbivores have often been historically lost from local species pools is clearly related to their large-body size (> 10 kg) as noted elsewhere^62^. Defaunation in clades including a small number of species is expected to be easily inflated. However, our metric of defaunation was largely invariant across mammalian orders (DI range = 0.61–0.67) given that speciose and widespread orders or those containing many secretive species were skewed to low values, suggesting that our estimates of local species loss were fairly accurate across the New World tropics.

The Atlantic Forest and Caatinga biomes succumbed to an average loss of 62% and 75% of their mammal faunas against their relatively recent historical baseline. Removing these two regions, the average defaunation across the entire Neotropics decreases by 20% from DI_adj_ = 0.56 to DI_adj._ = 0.45. These two biomes have a ~ 500-yrs history of deforestation and chronic overhunting since the early colonial era^63^, which has wiped out a substantial fraction of the original local mammal diversity, with the most representative assemblages currently confined to a few large core remnants^64,65^. On the order hand, the Pantanal wetlands experienced the lowest level of defaunation among all Neotropical biomes (DI_adj._ = 0.34), largely because land-use economics in this region has so far relied heavily on low-yield, extensive cattle ranching. Together with the Amazon and Patagonia, the Pantanal hosts the most intact contemporary mammal faunas^66,67^, not least because these remain the most structurally and faunally intact regions in the American tropics^68,69^. However, this encouraging situation can change rapidly in decades to come as global markets and lucrative land-based investments are expected to drive rapid agricultural frontier expansion^70^.

Our results reinforce the quintessential role of formal protected areas, where overall defaunation (DI_adj_) on average was 18.3% lower than at sites in unprotected landscapes. In our dataset, 653 (63.5%) of all sites experienced DI_adj_ greater than 50%, but 507 of those (77.6%) were unprotected sites. The most faunally-intact sites (DI_adj._ ≤ 0.30) included biological reserves, protected wildlife corridors, national and state forest reserves, and indigenous territories. The critical importance of protected areas in retaining biodiversity has been widely recognized^71^. Depending on location, protected areas retain high levels of native vegetation cover^72^, often substantially increasing species richness and abundance^73^, and can serve as a safety-net even in the most mammal-defaunated regions of South America^6^. However, the challenge to augment connectivity in conservation landscapes integrating protected and unprotected areas has become increasingly critical^72^, given that the latter can support over 50% of all biotic lineages^74^. Indigenous territories also play a key conservation role, often performing even better than conventional nature reserves in retaining primary habitat^75^. Large indigenous reserves, particularly in Amazonia, are also very sparsely settled^76^, often ensuring low hunting pressure on large mammals and a healthy landscape-scale source-sink dynamics in which even the most harvest-sensitive game species can thrive^77^.

All 32 Neotropical ungulate species were expected to be present 5,016 times across all 1,029 mammal assemblages, but were actually recorded only 1,733 times (34.6%). Among these species, lowland tapir was missing from 62.3% of all sites where they once occurred. The absence of ungulates can lead to a collapse in many ecological processes, such as herbivory, seed dispersal, seed predation, ecosystem landscaping^20,23^, and as important natural prey for apex-predators. These local extirpations are usually related to the legal jurisdiction (e.g., poorly enforced protected areas), landscape context (e.g., sparse habitat area), and elevated hunting pressure. Our results at the 4.5-km buffer scale show that the magnitude of defaunation was reduced at sites where natural vegetation cover was higher than 70% (DI_adj._ = 0.50), compared to sites where habitat area < 70% (DI_adj._ = 0.64). Nevertheless, defaunation rates were elevated even in areas of vast primary habitat, reinforcing the notion of “empty forests”^18^, which has been vindicated by several empirical studies (e.g.,^6,8^). Defaunation was less severe in more intact landscapes, decreasing to an average of 29.7% at 306 sites where habitat area exceeded 90%. Our results reinforce the importance of habitat area, which explained 21% of the variance in defaunation. Indeed, the synergistic effects of habitat area and hunting pressure accounted for 38% of the overall defaunation across the Neotropics.

We also show significant downsizing of Neotropical mammal assemblages, which was clearly associated with heavily hunted sites, particularly in landscapes where native vegetation had been converted to other land-uses. For instance, the size structure of assemblages at hunted sites exposed to a HPI > 0.40 was downsized by – 12.8 ± 6.4 kg, whereas sites under low hunting pressure (HPI < 0.40) were downsized by – 8.5 ± 5.7 kg. Hunting pressure and habitat loss explained 50% of variation in assemblage downsizing. Contrary to our expectations, the human footprint index (HFI) was a poor predictor of defaunation, but significantly explained the degree of assemblage downsizing.

Our continental-wide spatial interpolation shows that only 2.1% and 1.6% of the entire Neotropics had been defaunated by less than 25% and more than 75% in terms of local species losses, respectively. This compares with ~ 45% of the total area defaunated by 25–50%, and another ~ 45% defaunated by 50–75%. This is at odds with a recent global-scale study using similar predictors (e.g., HFI) as a proxy of hunting pressure, which estimates defaunation levels lower than 20% for ~ 85% of the entire Neotropics^78^. Here, we show that over 95% of the ~ 20 million km^2^ Neotropical realm had been defaunated by well over 20%. These divergent results can be partly resolved by simply disentangling the cause-effect relationships between hunting pressure, other environmental stressors and mammal diversity.

The HFI is a synthetic snapshot proxy of human impacts on ecosystems based on human population density, accessibility, infrastructure, land use change, and attempts to depict geographic gradients of overall human disturbance^79,80^, but is not necessarily a good predictor of hunting pressure. This is consistent with another global-scale analysis showing that HFI is only poorly correlated with mammal assemblage intactness^81^. However, there is a profound mismatch between the projection by Belote et al.^81^ of assemblage intactness and our continental-scale interpolation based on empirical evidence. Even assuming an overall 20% rate of false-presences in our historical assemblages—a value used to assess mismatches and omission errors from IUCN polygons for cryptic Mesoamerican amphibians^82^—our estimates of adjusted defaunation would only decrease from 56.5 to 45.2%. This conservative value is only 7.8% lower than our interpolated mean adjusted defaunation (49%). We submit that our Neotropics-wide defaunation estimates (ranging from 45.2 to 56.5%) are highly credible given the evidence to date (see Fig. 3B), particularly considering that IUCN polygons are sufficiently robust for macroecological analyses of relatively well sampled taxa, such as large mammals^36^. Recent global-scale estimates of defaunation therefore appear to be unduly optimistic, especially for high-degraded regions such as the semi-arid Caatinga and the Atlantic Forest.

Widely available recent spatial-layers representing overall human disturbance (e.g., HFI) have been used as spatial proxies of hunting pressure. However, these layers primarily represent land-use change, rather than direct wildlife population depletion, which is not necessarily correlated with habitat loss and degradation. Hunting offtakes per se may therefore often become a secondary factor in aggregate-disturbance measures of defaunation. Our HPI metric was a poor correlate of other predictors (covariance = – 0.153 ± 0.25), but significantly influenced assemblage downsizing. The HPI metric proposed here, which otherwise cannot be easily measured in situ, is therefore a promising proxy of local hunting pressure that can describe the impact of hunting on vertebrate populations and assemblages at very large scales.

Other anthropogenic disturbances also affect mammal guild structure and trophic organization (e.g.,^10,83–85^). Although accounting for only weak explanatory power (e.g., 6%; *p* = 0.04), high-intensity fire disturbance (> 200 hotpixels per 1-km grid cell) on average amplified the level of defaunation by ~ 10%. Forest wildfires trigger a cascade of detrimental effects on biodiversity, particularly in areas that experienced little or no fire-stress at an evolutionary timescale, leading to wholesale changes in species turnover^86,87^. This is particularly the case of Amazonia, which harbours the highest mammal alpha diversity, where wildfires are becoming more frequent and more intensive in all seasonally dry areas.

Presumed local extinctions were highly nonrandom with respect to mammal life-history traits and their ecological roles in the ecosystem. For instance, the body mass distribution of local mammal assemblages was greatly reduced from a historical 95th-percentile of ~ 14 kg to only ~ 4 kg in modern assemblages. Our assessment of environmental predictors showed that this was largely attributed to hunting pressure, and landscape-scale habitat area, habitat degradation and land-use change. This modern wave of extirpations is above and beyond that of the Pleistocene overkill by paleo-hunters which selectively eliminated many gigantic representatives of at least 12 major Neotropical mammal clades^88^ following the Great American Interchange of both megafauna and humans^89^. Our mechanistic understanding of how vertebrate body size responds to human threats remains at best imperfect. Body size may be central to the ecomorphological weaponry conferring adaptive tolerance to habitat change over evolutionary time (e.g.,^90–92^), whereas others argue that it is a key trait predisposing species susceptibility to environmental stressors (e.g.,^93,94^). Large-bodied species are often severely depleted by widespread conversion of native habitats into croplands and exotic pastures (e.g.,^95–97^). This disproportionate purge of large-bodied species is accompanied by detrimental consequences to trophic interactions, including both top-down and bottom-up processes^98–100^.

We also highlight the alarming shrinkage of local primate faunas (DI = 0.62). Neotropical primates are highly arboreal and forest-dependent, exerting pivotal roles in forest phytodemographic dynamics through effective seed dispersal, which eventually reverberates into key ecosystem processes such as seed dispersal and carbon storage^9,101^. However, primate assemblages have been severely simplified worldwide^102^, which is clearly aggravated in both overhunted^9^ and structurally degraded forest landscapes^103^. The highest proportions of presumed primate extinctions occurred in Mesoamerica (DI = 0.68 ± 0.40), northwestern South America (DI = 0.67 ± 0.40), and Western-Ecuador (DI = 0.56 ± 0.41). These patterns of decline in local species pools were similar for carnivores and rodents (62%), and the only order that showed average defaunation values below 61% was the Didelphimorphia.

Patterns of species deletions were largely consistent among, rather than within, mammal orders. For example, presumed extinction in bush dogs (*Speothus venaticus*) were the most ubiquitous across all carnivores, but large-bodied species were among the most frequently extirpated mammals. The two large felid apex-predators—*Panthera onca* and *Puma concolor*—were similarly presumed extinct in over half (53.7%) of all assemblages. The low overall level of observed carnivore defaunation is due to a prevalence of smaller-bodied mesopredator species that could benefit from the absence of large carnivores. However, extant large carnivore populations often persist in human-modified landscapes at extremely low densities, where they no longer perform their ecological roles^104^. In contrast, several widespread, habitat-generalist species (e.g., *Mazama gouazoubira*, *Pecari tajacu*, *Nasua nasua*, *Leopardus pardalis*, *Cerdocyon thous*, *Eira barbara*) on average failed to occur in 48.0% of all assemblages (range = 31.3–61.4%).

Although our confusion matrix adjustment minimizes the likelihood of defaunation overestimates, we suggest more survey efforts targeting rare, habitat-specialist, or otherwise inconspicuous species that are inherently prone to false-absences using conventional methods (but see^105^). We acknowledge that a standardized intensive sampling program would likely reduce the likelihood of false-absences, which artificially inflate defaunation estimates, but this is unlikely to be implemented at a continental scale in the foreseeable future. Our interpolation of mammal species loss confirms regional scale assessments in the Atlantic Forest (e.g.,^6,50^). However, we detected some quite severe interpolation failures due to poor data resolution inducing Wallacean shortfalls, such as in northern Amazonia (see Fig. 1). These shortfalls add to the challenges of expanding large-scale biodiversity knowledge in the most species-rich biomes, which are most prone to gargantuan Linnean and Wallacean shortfalls^106^. We therefore acknowledge any interpolation can be severely biased against large undersampled areas. Yet these estimates are also enhanced by faunally-intact sites that have been comprehensively inventoried, such as Cocha Cashu^107^ and Urucu^108^ in southwestern and central Amazonia, respectively.

This study vindicates our initial working hypotheses that (1) historical defaunation is a pervasive phenomenon throughout the Neotropical realm, but far more severe in regions that succumbed to widespread primary habitat conversion and degradation to agriculture in the early post-colonial period. Notwithstanding, many mammal populations in more intact areas have also been driven to local extinctions through a combination of overhunting and drivers of habitat degradation, such as wildfires; (2) degree of local defaunation and downsizing of species in residual assemblages were aggravated by land-use pressures, overhunting, and to a lesser extent other measures of the human disturbance footprint, which indicate that the detrimental effects on mammal community decay is size-biased and depends on key life history traits; (3) landscape-scale retention of large core-areas of native primary habitat buffers the intensity of defaunation, even though structurally intact landscapes are not immune to species losses if they have been persistently overhunted; and (4) ungulates are among the most heavily extirpated mammal orders, whereas most carnivores species persist to a surprisingly high degree, not least because non-threatened carnivores are most likely to use anthropogenic matrix habitats^109,110^.

Our findings—based on a comprehensive compilation of 1,029 spatially independent mammal assemblages spanning ~ 10,700 km and 85° of latitude across 23 countries—can be used to inform international conservation policies to prevent further erosion of, if not restore, native biodiversity. Further conservation efforts should be mobilized to prevent the most faunally-intact biomes, such as Amazonia and the Pantanal wetlands, from following in the footsteps of “empty ecosystems” that are now typical of historically degraded biomes such as the Atlantic Forest and the Caatinga. This includes de facto implementation and law enforcement in existing protected areas, and thwarting political pressures to either downgrade or downsize these areas. In several cases we still need to identify new protected areas that that—despite the aforementioned caveats—can be highly effective in protecting native biotas^72–74^.

We further encourage realistic efforts to rewild the most chronically defaunated Neotropical regions, prioritizing areas that may strategically serve as population sources to adjacent areas, such as in the severely defaunated Northern Atlantic Forest. Fortunately, several of these areas coincide with economically affluent regions, such as southeastern Brazil, that can afford to both protect wildlife from overhunting and enhance connectivity in otherwise highly fragmented landscapes through dispersal corridors and new habitat set-asides. Greater investment should be allocated to more effective control of illegal hunting (particularly focusing on commercial hunting), deforestation, and anthropogenic fires, as well as ensure the legally expected *status quo* of fully implemented protected areas. Finally, sound resource management should be sensitive to socioeconomic context, while recruiting rather than antagonizing potential local alliances who can effectively fill the institutional void in low-governance regions. Hominins and other mammals have coexisted since the earliest Paleolithic hunters wielding stone tools some 3–4 million years ago. Over this long timescale biodiversity losses have only recently accelerated to breakneck speeds since the industrial revolution. Let us make sure that most of this impoverishment is behind rather than ahead of us, or else the prospects for Neotropical mammals will look increasingly bleak.

## Supplementary information

Supplementary Legends.

Supporting Information S1.

Supporting Information S2.

Supporting Information S3.

Supporting Information S4.

Supporting Information S5.

Supporting Information S6.

Supporting Information S7.

Supporting Information S8.
