# Supplementary material for: Extent, intensity and drivers of mammal defaunation: a continental-scale analysis across the Neotropics
Source: Sci Rep. 2020 Sep 15;10:14750. doi: 10.1038/s41598-020-72010-w (PMC7492218; doi:10.1038/s41598-020-72010-w)
Supplement: Supplementary file 1 — Supplementary Legends. [file 41598_2020_72010_MOESM1_ESM.docx]

**Bogoni et al. — Extent, intensity and drivers of mammal defaunation: a continental-scale analysis across the Neotropics**

**Supporting Information legends**

**Supporting Information S1.** Checklist of all references used to compile the 1,029 mammal assemblages across the Neotropical realm.

**Supporting Information S2.** Theoretical models to compose the hunting pressure index (HPI) across the Neotropical realm.

**Supporting Information S3.** References and links to sources of data layers used to assemble the hunting pressure index (HPI).

**Supporting Information S4.** Example of hunting pressure index (HPI) calculation across the Neotropical realm.

**Supporting Information S5.** Application of Bayes Theorem to the hunting pressure index (HPI).

**Supporting Information S6.** Interpolation of hunting pressure index (HPI) across the Neotropical realm.

**Supporting Information S7.** (A) Interpolation of Bayes-posterior probability to all values derived from Eq. 1 vs. Eq. 2 across the Neotropical realm (HPI evaluation); and (B) Interpolation of false-absence ratio across the Neotropical realm boundaries (defaunation bias correction).

**Supporting Information S8.** Interpolation of adjusted defaunation across the Neotropical realm.
