# Supplementary material for: Extent, intensity and drivers of mammal defaunation: a continental-scale analysis across the Neotropics
Source: Sci Rep. 2020 Sep 15;10:14750. doi: 10.1038/s41598-020-72010-w (PMC7492218; doi:10.1038/s41598-020-72010-w)
Supplement: Supplementary file 2 — Supporting Information S1. [file 41598_2020_72010_MOESM2_ESM.docx]

**Supporting Information S1.** Checklist of all references used to compile the 1,029 mammal assemblages across the Neotropical realm.

Abreu-Júnior, E.F. and Köhler, A. (2009). Mammalian fauna of medium and large sized in the RPPN of UNISC, RS, Brazil. Biota Neotrop. 9, 169–174.

Acosta, L., Aguanta, F.A. 2006. Lista preliminar de los mamíferos del bosque experimental Elías Meneses, Santa Cruz, Bolivia. Kempffiana 2(1):144-149.

Aguilar-López, et al. 2013. Lista taxonómica y estructura del ensamblaje de los mamíferos terrestres del municipio de Tlanchinol, Hidalgo, México. Mastozoología Neotropical 20(2): 229-242.

Albanesi, S.A., et al. 2019. Mamíferos De Mediano Y Gran Porte En Corredores Boscosos Del Pedemonte De Yungas Del Noroeste Argentino. Mastozoología Neotropical, in press.

Albuquerque, H.G., Martins, P.F., Pessôa, F.S., Carvalho, T., Modesto, T.C., Luz, J.J., Raíces, D.S.L., Ardete, N.C., Lessa, I.C.M., Attias, N., Jordão-Nogueira, T., Enrici, M.C. and Bergallo, H.G. (2013). Mammals of a forest fragment in Cambuci municipality, state of Rio de Janeiro, Brazil. Check List. 9, 1505-1509.

Alvarenga, G.C., et al. 2018. Spatial patterns of medium and large size mammal assemblages in várzea and terra firme forests, Central Amazonia, Brazil. PLoS ONE 13(5): e0198120.

Alvarez-Castaneda, S.T., et al. 2008. Mamiferos de las Reservas de El Valle de los Cirios e El Vizcano. Primera ed., Universidade Autonoma Metropolitana.

Alves, G.B. et al. 2014. Medium and large-sized mammals of a fragment of Cerrado in the Triângulo Mineiro region, southeastern Brazil. Biosci. J. 30(3): 863-873.

Alves, T. R., Fonseca, R. C., and Engel, V. L. (2012). Mamíferos de médio e grande porte e sua relação com o mosaico de habitats na cuesta de Botucatu, Estado de São Paulo, Brasil. Iheringia. S. Zool. 102, 150-158.

Alves, T.R., Fonseca, R.C.B. and Engel, V.L. (2012). Medium and large sized mammalians and their relation to habitat patches at the Botucatu cuesta, state of São Paulo, Brazil. Iheringia. Iheringia Sér. Zool. 102, 150-158.

Alves, V.N., et al. 2019. Medium and large-sized mammals in an Atlantic Forest fragment of Brazil: recording of threatened species Journal of Threatened Taxa 11(3): 13278–13286.

Andrade, M.J., Castro, J. 2009. Mamíferos grandes y medianos: relación con hábitat y paisaje María José Andrade y Jessica Castro. Uruguay Ciencia 2009: 26-29

Andrade, R.P., et al. 2019. Accessibility do not Explain Abundance of Medium and Large-Sized Mammals in Terra Do Meio, Altamira, Pará, Brazil. Acta Zool. Mexicana 35: 1–10.

Andrade-Núñez, M.J., Aide, M. 2010. Effects of habitat and landscape characteristics on medium and large mammal species richness and composition in northern Uruguay. Zoologia 27(6): 909–917.

Ángel, A.Q. 2011. Riqueza Y Composición De Mamíferos Grandes Y Medianos De Cinco Localidades Del Valle Del Cauca Reisa Nadiesdha Valencia Daza. Undergraduate Thesis, Universidad Del Valle.

Anleu, B.I.E. 2015. Riqueza De Mamíferos Medianos Y Mayores En Cafetales Y Bosques De Tres Reservas Naturales Privadas (San Jerónimo Miramar-Quixayá, Pampojilá-Peña Flor Y Santo Tomás Pachuj) De La Reserva De Usos Múltiples De La Cuenca Del Lago De Atitlán –Rumcla. Undergraduate thesis, Universidad De San Carlos De Guatemala.

Aranda, M., et al. 2012. Diversidad y datos reproductivos de mamíferos medianos y grandes en el bosque mesófilo de montaña de la Reserva de la Biosfera Sierra de Manantlán, Jalisco- Colima, México. Revista Mexicana de Biodiversidad 83: 778-784.

Araújo, G.R., et al. 2015. Mamíferos de médio e grande porte em um fragmento florestal de Cerrado no município de Ipameri-GO. Multi-Science Journal 1(1): 55-61

Asquith, N., Mejía-Chang, M. 2005. Mammals, Edge Effects, and the Loss of Tropical Forest Diversity. Ecology 86(2): 379–390.

Azcárraga, A.A. 2013. Riqueza y la abundancia de mamíferos medianos de la reserva biológica Tirimbina, Costa Rica. Therya 4(3): 597-601.

Bastazini, V.A.G. 2011. Efeitos da estrutura de habitat e do espaço sobre a diversidade de mamíferos no norte do Pantanal: uma abordagem de resolução fina. PhD Thesis, Universidade Federal do Rio Grande do Sul.

Behnke, R. 2015. A camera-trap based inventory to assess species composition of large- and medium-sized terrestrial mammals in a Lowland Amazonian rainforest in Loreto, Peru: a comparison of wet and dry season. Mater thesis, University of Natural Resources and Life Sciences (BOKU) in Vienna.

Benchimol, M., Peres, C.A. 2015. Predicting local extinctions of Amazonian vertebrates in forest islands created by a mega dam. Biological Conservation 187: 61–72.

Bergós, L., et al. 2018. Fogones de Fauna: An Experience of Participatory Monitoring of Wildlife in Rural Uruguay. Society & Animals 26: 171-185.

Bermúdez-Enríquez, O., et al. 2013. Medium and Large Mammals in the Sierra La Madera, Sonora, Mexico. Forest Service Proceedings 67: 463-469.

Bernardo, P.V.S., Melo, F.R. 2013. Assemblage of medium and large size mammals in an urban Semideciduous Seasonal Forest fragment in Cerrado biome. Biota Neotrop. 3(2). Doi: 10.1590/S1676-06032013000200008.

Bianchin, J.F., Koenemann, J.G., Chiva, E.Q. 2011. Mamíferos não voadores encontrados em três áreas do Parque Estadual do Espinilho, Barra do Quaraí, Rio Grande do Sul. Biodiversidade Pampeana 9(1): 44-49.

Bisbal, F.J., Rivero, R. 2005. Notas sobre los vertebrados de la isla de Cubagua, Venezuela. Memoria de la Fundación La Salle de Ciencias Naturales 163: 5-17.

Blake, J.G., et al. 2012. Temporal activity patterns of terrestrial mammals in lowland rainforest of eastern Ecuador. Ecotropica 137(18): 137-146

Blake, J.G., Loiselle, B.A. 2018. Annual and spatial variation in composition and activity of terrestrial mammals on two replicate plots in lowland forest of eastern Ecuador. PeerJ 6: e4241.

Blake, J.G., Morquera, D., Salvador, J. 2012. Use of mineral licks by mammals and birds in hunted and non-hunted areas of Yasuní National Park, Ecuador. Animal Conservation 16(4). Doi: 10.1111/acv.12012.

Bocchiglieri, A., Mendonça, A.F., Henriques, R.P.B. 2010. Composition and diversity of medium and large size mammals in the Cerrado of central Brazil. Biota Neotrop. 10(3): 10.

Bodmer, R, Fang, T., Puertas, P., Durrell Institute of Conservation and Ecology. 2009. Wildlife Populations in the Pacaya-Samiria National Reserve and Lago Preto, Peru Report for Operation Wallacea. National University of the Peruvian Amazon (UNAP), National Institute of Natural Resources – Peru (INRENA), and Pacaya-Samiria National Reserve Authority.

Bogoni, J.A., Bogoni, T.C., Graipel, M.E. and Marinho, J.R. (2013). The Influence of Landscape and Microhabitat on the Diversity of Large-and Medium-Sized Mammals in Atlantic Forest Remnants in a Matrix of Agroecosystem and Silviculture. ISRN Forestry 2013.

Bogoni, J.A., Cherem, J.J., Giehl, E.L.H., Oliveira-Santos, L.G.R., Castilho, P.V., Picinatto-Filho, V., Fanticini, F.M., Tortato, M.A., Luiz, M.R., Rizzaro, R. and Graipel, M.E. (2016a). Landscape features lead to shifts in communities of medium to large-bodied mammals in subtropical Atlantic Forest. J. Mammal., gyv215.in press.

Bogoni, J.A., et al. (unpublished data).

Bogoni, J.A., et al. (unpublished data).

Bogoni, J.A., et al. (unpublished data).

Bogoni, J.A., et al. 2017. What would be the diversity patterns of medium- to large-bodied mammals if the fragmented Atlantic Forest was a large metacommunity? Biological Conservation 211: 85-94.

Bogoni, J.A., Graipel, M.E., Castilho, P.V., Fantacini, F.M., Kuhnen, V.V., Luiz, M.R., Maccarini, T.B., Marcon, C.B., Teixeira, C.S.P., Tortato, M.A., Vaz-de-Mello, F.Z., Hernández, M.I.M (2016b). Contributions of the mammal community, habitat structure, and spatial distance to dung beetle community structure. Biod. Cons. 25, 1661-1675.

Bonino, N. 2005. Guía de Mamíferos de la Patagonia Argentina. Instituto Nacional de Tecnología Agropecuária. Erregé & Associados.

Borges, L.H.M., et al. 2014. Diversity and habitat preference of medium and large-sized mammals in an urban forest fragment of southwestern Amazon. Iheringia Série Zoologia Doi: 10.1590/1678-476620141042168174.

Borges, L.H.M., et al. 2015. Large and Medium-Sized Mammals from Chandless State Park, Acre, Brazil. Mastozoología Neotropical 22(2): 265-277.

Boron, V. et al. 2019. Richness, diversity, and factors influencing occupancy of mammal communities across human-modified landscapes in Colombia. Biological Conservation 232: 108–116.

Borroto-Páez, R., Mancina, C.A. 2017. Biodiversity and conservation of Cuban mammals: past, present, and invasive species. Journal of Mammalogy 98(4):964–985.

Botelho, A.L.M., et al. 2012. Large and medium-sized mammals of the Humaitá Forest Reserve, southwestern Amazonia, state of Acre, Brazil. Check List 8(6): 1190–1195.

Bovendorp, R. S. and Galetti, M. (2007). Density and population size of mammals introduced on a land-bridge island in southeastern Brazil. Biol. Invasions 9, 353-357.

Briones-Salas, M., Lavariega, M. C., Lira-Torres, I., 2018. Mammal diversity before the construction of a hydroelectric power dam in southern Mexico. Animal Biodiversity and Conservation 42.1: 99–112.

Bristot, T.C. 2013. Levantamento de mamíferos de médio e grande porte em área de Cerrado, no oeste goiano. Master thesis, Universidade Católica de Goiás.

Brito, J.M., Ojala-Barbour, R. 2016. Mamíferos no voladores del Parque Nacional Sangay, Ecuador. Papeis Avulsos de Zoologia 56(5): 45‑61.

Brocardo, C.R. and Cândido-Júnior, J.F. (2012). Persistência de mamíferos de médio e grande porte em fragmentos de Floresta Ombrófila Mista no estado do Paraná, Brasil. Rev. Árvore 36, 301-310.

Brown, M.T., et al. 2006. Aquatic Sciences Overview Article Species diversity in the Florida Everglades, USA: A systems approach to calculating biodiversity. Aquat. Sci. 68: 254–277.

Buenostro-Silva, Antonio-Gutiérrez, M., García-Grajales, J. 2012. Mamíferos Del Parque Nacional Lagunas De Chacahua Y La Tuza De Monroy, Oaxaca, México. Acta Zoológica Mexicana 28(1): 56-72.

Cabral, R. et al. 2017. Medium-sized to large mammals of Serra do Tombador, Cerrado of Brazil. Check List 13(3): 2129.

Cáceres, N.C. et al. 2007. Mammals of the Bodoquena Mountains, Mammals of the Bodoquena Mountains, southwestern Brazil: an ecological and conservation analation analysis. Revista Brasileira de Zoologia 24 (2): 426–435.

Cáceres-Martínez, C.H., et al. 2016. Terrestrial medium and large-sized mammal’s diversity and activity patterns from Tamá National Natural Park and buffer zone, Colombia. Therya 7(2): 285-298.

Calaça, A., et al. 2019. Mammals recorded in isolated remnants of Atlantic Forest in southern Goiás, Brazil. Biota Neotrop. 19(1). Doi: 10.1590/1676-0611-bn-2018-0575.

Calouro, A.M. 1999. Riqueza de mamíferos de grande e médio porte do Parque Nacional da Serra do Divisor (Acre, Brasil). Rev. Bras. Zool. 16(2): 195-213.

Câmara, E.M.V.C., Oliveira, L.C. 2012. Mammals of Serra do Cipó National Park, southeastern Brazil. Check List 8(2): 355-359.

Campos, C.B., et al. 2019. Medium and large sized mammals of the Boqueirão da Onça, North of Bahia State, Brazil. Pap. Avulsos Zool. (59): e20195912.

Campos, F.S., et al. 2013. Diversity of medium and large sized mammals in a Cerrado fragment of central Brazil. Journal of Threatened Taxa 5(15): 4994-5001.

Campos, V.E. et al. 2017. Richness of plants, birds and mammals under the canopy of Ramorinoa girolae, an endemic and vulnerable desert tree species. Bosque 38(2): 307-316.

Canale, G., et al. 2016. Mamíferos de médio e grande porte. In: Anjos-Silva, E. 2016. Biodiversidade do Parque Estadual Cristalino. Áttema Editorial.

Canale, G.R., Peres, C.A., Guidorizzi, C.E., Gatto, C.A.F., Kierulff, M.C.M., 2012. Pervasive defaunation of forest remnants in a tropical biodiversity hotspot. PLoS ONE 7: e41671.

Cançado, H.D., et al. 2017. Current status of ticks and tick-host relationship in domestic and wild animals from Pantanal wetlands in the state of Mato Grosso do Sul, Brazil. Iheringia Série Zoologia 107(supl.): e2017110.

Carmignotto, A.P., Aires, C.C. 2011. Mamíferos não voadores (Mammalia) da Estação Ecológica Serra Geral do Tocantins. Biota Neotrop. 11(1): 313-328.

Caro, T.M., et al. 2001. Inventorying Mammals at Multiple Sites in the Maya Mountains of Belize. Journal of Mammalogy 82(1): 43-50.

Carvalho I.D., Oliveira R. and Pires A.S. (2014) Medium and large-sized mammals of the Reserva Ecológica de Guapiaçú, Cachoeiras de Macacu, RJ. Biota Neotrop. 14, 1–9.

Carvalho, A.S., et al. 2014. Large and Medium-Sized Mammals of Carajás National Forest, Pará State, Brazil. Check List 10(1): 1–9.

Carvalho, W.D., Godoy, M.S.M., Adania, C.H. and Esbérard, C.E.L. (2013). Non-volant mammal assemblage of serra do Japi biological reserve, Jundiaí, São Paulo, southeastern Brazil. Bioscience J. 29, 1370-187.

Cassano, C.R., Barlow, J. and Pardini, R. (2012). Large mammals in an agroforestry mosaic in the Brazilian Atlantic Forest. Biotropica 44, 818-825.

Castañeda, F., et al. 2013. Diversidad de mamíferos terrestres en fincas de cacao de Olancho, Honduras. Reporte. Panthera/Helvetas-Honduras/Aprosacao.

Castillo-Figueroa, D. et al. 2019. Structural differences in mammal assemblages between savanna ecosystems of the Colombian Llanos. Pap. Avulsos Zool. 59: e20195914.

Castro, W.J.P. 2015. Probabilidade De Ocupação De Manchas Florestais Por Médios E Grandes Mamíferos Na Sub-Região Da Nhecolândia, Pantanal, Mato Grosso Do Sul, Brasil. Master thesis, Universidade Federal de Mato Grosso do Sul.

Centro de Desarollo Agrario y Florestal. 2001. Diagnóstico e inventário de los recursos naturales de flora y fauna. Linderos de la Reserva Nacional del Titicaca. Informe Final. CIRNMA, CEDAFOR-Perú.

Cervera, L. et al. 2016. A camera trap assessment of terrestrial mammals in Machalilla National Park, western Ecuador. Check List 12(2): 1868.

Chagas, R. R. D., Junior, E. M. S., Souza-Alves, J. P. and Ferrari, S. F. (2011). Fazenda Trapsa, um refúgio de diversidade de mamíferos de médio e grande porte em Sergipe, Nordeste do Brasil. Rev. Nordestina. Biol. 19, 35-43.Chagas.et.al.2012

Charre-Medellín, J. F., et al. 2016. Mamíferos medianos y grandes del municipio de Victoria, Reserva de la Biosfera Sierra Gorda Guanajuato, México. Acta Universitaria, 26(2): 62-70.

Cherem, J.J. and Perez, D.M. (1996). Mamíferos terrestres de floresta de araucária no município de Três Barras, Santa Catarina, Brasil. Biotemas 9, 29-46.

Cherem, J.J., Graipel, M.E., Tortato, M.A., Althoff, S.L., Brüggemann, F., Matos, J.Z., Voltolini, J.C., Freitas, R.R., Illenseer, R., Hoffmann, F., Ghizoni-Jr., I.R., Bevilacqua, A., Reinicke, R., Oliveira, C.H.S., Filippini, A., Furnari, N., Abati, K., Moraes, M., Moreira, T.T., Oliveira-Santos, L.G.R., Kuhnen, V.V., Maccarini, T.B., Goulart, F.V.B., Mozerle, H.B., Fantacini, F.M., Dias, D., Penedo-Ferreira, R., Vieira, B.P. and Simões-Lopes, P.C. (2011). Mastofauna terrestre do Parque Estadual da Serra do Tabuleiro, Estado de Santa Catarina, sul do Brasil. Biotemas 24, 73-84.

Chiarello, A.G. (1999). Effects of fragmentation of the Atlantic forest on mammal communities in South-eastern Brazil. Biol. Conserv. 89, 71–82.

Coronel-Arellano, H. et al. 2016. Species richness and conservation status of medium and large terrestrial mammals from four Sky Islands in Sonora, northwestern Mexico. Check List 12(1): 1839.

Cortés-Marcial, M., Briones-Salas, M. 2014. Diversidad, abundancia relativa y patrones de actividad de mamíferos medianos y grandes en una selva seca del Istmo de Tehuantepec, Oaxaca, México. Rev. Biol. Trop. 62(4): 1433-1448.

Costa, H.C.M., et al. 2018. Seasonal dynamics of terrestrial vertebrate abundance between Amazonian flooded and unflooded forests. PeerJ 6: e5058. Doi: 10.7717/peerj.5058.

Covel, M.V., et al. 2013. Integrating occupancy modeling and camera-trap data to estimate medium and large mammal detection and richness in a Central American biological corridor. Tropical Conservation Science 6(6): 781-795.

Cruz-Bazán, E.J. et al. 2017. Diversidad de mamíferos terrestres en una área privada de conservación en en Talhpan, Veracruz, Mexico. Esosist. Recur. Agropec. 4(10):123-133.

Cruz-Jácome, O., et al. 2015. Richness and relative abundance of medium and large mammals in a community of the Biosphere Reserve Tehuacán-Cuicatlán, Oaxaca, Mexico. Therya 6(2): 435-448.

Cullen Jr., L., Bodmer, R.E. and Valladares-Pádua, C. (2001). Ecological consequences of hunting in Atlantic forest patches, São Paulo, Brazil. Oryx 35, 137–144.

Cunha, A. A. (2010). Negative effects of tourism in a Brazilian Atlantic Forest National Park. J. Nat. Conserv. 18, 291-295.

Cunha, A.A., and Rajão, H. (2007). Mamíferos Terrestres e Aves da Terra Indígena Sapukai (Aldeia Guarani do Bracui), Angra dos reis, Rj, Brasil. Bol. Mus. Biol. Mello Leitão 21, 19-34.

D’Agostino, R.L., Sauthier, D.U. 2017. Los hay pequeños, medianos y grandes: mamíferos terrestres. In: Sauthier, D.U. 2017. Reserva de Vida Silvestre San Pablo de Valdés, 10 años conservando el patrimonio natural y cultural de la Península Valdés, Patagonia Argentina. FVSA-CONICET. Pp: 35-45.

Dalecky, A. et al. 2002. Large Mammals on Small Islands: Short Term Effects Of Forest Fragmentation On The Large Mammal Fauna In French Guiana. Rev. Écot. (Terre Vie) 57: 145-165

Dario, F.R. 2018. Traditional knowledge of the wild mammals and their ecological interactions by community indigenous Apiaká, Southern Brazilian Amazon Rainforest. World News of Natural Sciences 17: 48-55.

Delciellos, A.C. 2016. Mammals of four Caatinga areas in northeastern Brazil: inventory, species biology, and community structure Ana Cláudia Delciellos Check List 12(3): 1916.

Delciellos, A.C., Novaes, R.L.M., Loguercio, M.F.C., Geise, L., Santori, R.T., Souza, R.F.S., Papi, B.S., Raíces, D., Vieira, N.R., Feliz, S., Detogne, N., Silva, C.C.S., Bergallo, H.G. and Rocha-Barbosa, O. (2012). Mammals of Serra da Bocaina National Park, state of Rio de Janeiro, southeastern Brazil. Check List 8, 675-692.

Delibes-Mateos, M., et al. 2014. Caracterización de la comunidad de mamíferos de un área remota del sur de Chile mediante el uso combinado de metodologias. Galemys, 26: 65-75.

Desbiez, A.L.J., Bodmer, R.E., Tomas, W.M. 2010. Mammalian Densities in a Neotropical Wetland Subject to Extreme Climatic Events. Biotropica 42(3): 372–378.

Di Bitetti, M.S., Paviolo, A. and De Angelo, C. (2014). Camera trap photographic rates on roads vs. off roads: location does matter. Mastozool. Neotrop. 21, 37-46.

Dias, D.M. 2014. Mamíferos de médio e grande porte em uma área de Caatinga de Sergipe e o nicho ecológico de Cerdocyon thous (Linnaeus, 1766). Master thesis, Universidade Federal de Sergipe.

Dias, D.M., Bocchiglieri, A. 2016. Riqueza e uso do habitat por mamíferos de médio e grande porte na Caatinga, nordeste do Brasil Richness and habitat use by medium and large size mammals in Caatinga, northeastern Brazil. Neotropical Biology and Conservation 11(1): 38-46.

Dias, D.M., et al. 2017. Diversity of nonvolant mammals in a Caatinga area in northeastern Brazil Diversidade de mamíferos não voadores em uma área de Caatinga do nordeste do Brasil. Biology and Conservation 12(3): 200-208.

Dias, T.D. 2013. Comunidades de mamíferos de pequeno, médio e grande porte em fitofisionomias pampianas: diversidade e uso de habitat. Undergraduate thesis, Universidade Federal do Pampa.

Dias, W.A.F., Tezori, R.F.F. and Oliveira, A.K. (2012). Registro de mamíferos de médio e grande porte em dois fragmentos florestais no município de São Carlos, Estado de São Paulo. Multiciêcia 11, 277-293.

Díaz, I.A., et al. 2002. Vertebrados terrestres de la Reserva Nacional Río Clarillo, Chile central: representatividad y conservación Terrestrial vertebrates of the Río Clarillo National Reserve, central Chile: representation and conservation. Revista Chilena de Historia Natural 75: 433-448.

Dotta, G. and Verdade, L.M. (2011). Medium to large-sized mammals in agricultural landscapes of south-eastern Brazil. Mammalia 75, 345-352.

Duprat, P.L. and Andriolo, A. (2013). Mastofauna não-voadora de médio e grande porte em um fragmento de Mata Atlântica no município de Rio Novo, MG. Rev. Bras. Zoociênc. 13, 163-172.

Eduardo, A.A. (2011). Spatial patterns of mammalian diversity in a fragmented landscape in southeastern Brazil. Rev. Bras. Biociênc. 9, 252-255.

Espartosa, K.D., Pinotti, B.T. and Pardini, R. (2011). Performance of camera trapping and track counts for surveying large mammals in rainforest remnants. Biodiver. Conserv. 20, 2815-2829.

Espinossa, C.C., et al. 2016. Medium- and large-sized mammals in a steppic savanna area of the Brazilian Pampa: survey and conservation issues of a poorly known fauna. Braz. J. Biol. Doi: 10.1590/1519-6984.12714.

Estrela, D.C., et al. 2015. Medium and large-sized mammals in a Cerrado area of the state of Goiás, Brazil. Check List 11(4): 1690.

Falcão, F.C., Guanaes, D.H.A. and Paglia, A. (2012). Medium and large-sized mammals of RPPN Estação Veracel, southernmost Bahia, Brazil. Check List 8, 929-934.

Faúndez, P.V. 2012. Terrestrial mammals of the Atacama Region, Chile: Comments on Its distribution and conservation status. Gayana 76(1): 22-37.

Faustino, A.C. 2015. Estrutura da Comunidade de Mamíferos em Silvicultura de Teca (Tectona Grandis L. F.) no Cerrado, Pantanal e Amazônia em Mato Grosso, Brasil. Master thesis, Universidade do Estado de Mato Grosso.

Ferreguetti, A.C., et al. 2019. Medium- and large-sized mammal composition in the Chapada dos Veadeiros National Park and adjacent areas, state of Goiás, Brazil. Pap. Avulsos Zool. 59: e20195942.

Ferreira, H.F., et al. 2015. Non-volant mammals from Baturité Ridge, Ceará state, Northeast Brazil. Check List 11(3): 1630.

Flesher, K.M. and Laufer, J. (2013). Protecting wildlife in a heavily hunted biodiversity hotspot: a case study from the Atlantic Forest of Bahia, Brazil. Trop. Conserv. Sci. 6, 181-200.

Flores, A.G., et al. 2014. Uso de mamíferos silvestres por habitantes del Parque Nacional el Tepozteco, Morelos, México. Etnobiologia 12(3): 57-67.

Fornitano, L., Angeli, T., Costa, R.T., Olifiers, N. and Bianchi, R.C. (2015). Medium to large-sized mammals of the Augusto Ruschi Biological Reserve, São Paulo State, Brazil. Oecologia Oecol . Australis 19, 232-243.

Fracassi, N.G., et al. 2010. Nuevas especies de mamíferos para el bajo delta del Paraná y bajíos ribereños adyacentes, Buenos Aires, Argentina. Mastozoología Neotropical 17(2): 367-373.

Freitas, E.B., Carvalho, C.B., Ferrari, S.F. 2011. Abundance of Callicebus barbarabrownae (Hershkovitz 1990), (Primates: Pitheciidae) and other nonvolant mammals in a fragment of arboreal Caatinga in northeastern Brazil. Mammalia 75 (2011): 339–343.

Gajapersad, K. et al. 2012. A Survey of the Large Mammal Fauna of the Kwamalasamutu Region, Suriname. Conservation International. Doi: 10.1896/054.063.0115.

Gallina, S., González-Romero, A. 2018. Conservación La conservación de mamíferos medianos en dos reservas ecológicas privadas de Veracruz, México. Revista Mexicana de Biodiversidad 89: 1245-1254.

García, F.J., et al. 2012. Preliminary inventory of mammals from Yurubí National Park, Yaracuy, Venezuela with some comments on their natural history. Rev. Biol. Trop. 60 (1): 459-472.

García-Flores, A., et al. 2014. Uso De Mamíferos Silvestres Por Habitantes Del Parque Nacional El Tepozteco, Morelos, México. Etnobiología 12(3): 57-67.

Garmendia, A., et al. 2013. Landscape and patch attributes impacting medium and large sized terrestrial mammals in a fragmented rain forest. Journal of Tropical Ecology 29(4): 331-334.

Gatti, A., Ferreira, P.M., Cunha, C.J., Seibert, J.B., Moreira, D.O. 2017. Diversity of medium-sized and large mammals from Atlantic Forest remnants in southern Minas Gerais state, Brazil. Oecologia Australis 21(2): 171-181.

Gatti, A., Segatto, B., Carnelli, C.C. and Moreira, D.O. (2014). Mamíferos de médio e grande porte da Reserva Biológica Augusto Ruschi, Espírito Santo. Natureza on line 12, 61-68.

Geise, L., Pereira, L.G., Bossi, D.E.P. and Bergallo, H.G. (2004). Pattern of elevational distribution and richness of non volant mammals in Itatiaia National Park and its surroundings, in southeastern Brazil. Braz. J. Biol. 64, 599-612.

Gengler, N. 2018. Large-Medium Mammal Diversity in Fragmented Habitat: A Camera Trap Inventory Surrounding a Rural Community in the Upper Paraná Atlantic Forest of Paraguay. Bol. Mus. Nac. Hist. Nat. Parag. 22(2): 100-100.

Genoways, H., Timm, R.M. 2005. Mammals of the Cosigüina Peninsula of Nicaragua. Mastozoología Neotropical 12(2):153-179.

Gentry, A.H. 1993. Four Neotropical Rainforests. Yale University Press. 640p.

Giacomini, H.C. and Galetti, M. (2013). An index for defaunation. Biol. Conserv. 163, 33-41.

Gomes, L.P., Rocha, C.R., Brandão, R.A., Marinho Filho, J. 2015. Mammal richness and diversity in Serra do Facão region, Southeastern Goiás state, central Brazil. Biota Neotropica. 15(4): 1–11.

Gomes-Albuquerque, H., Martins, P.F., Pessôa, F.S., Modesto, T.C., Luz, J.L., Raíces, D.S., Ardente, N.C., Lessa, I.C.M, Attias, N., Jordão-Nogueira, T., Enrici, M. C. and Bergallo, H.G. (2013). Mammals of a forest fragment in Cambuci municipality, state of Rio de Janeiro, Brazil. Check List 9, 1505-1509.

Gómez, H., et al. 2001. Diversidad y abundancia de mamíferos medianos y grandes en el noreste del área de influencia del Parque Nacional Madidi durante la época húmeda. Ecología en Bolívia 36: 17-29.

Gonthier, D.J., Castañeda, F.E. 2013. Large- and medium-sized mammal survey using camera traps in the Sikre River in the Río Plátano Biosphere Reserve, Honduras. Tropical Conservation Science 6 (4): 584-591.

Gordillo-Chávez, E.J., et al. 2015. Mastofauna del humedal Chaschoc-Sejá en Tabasco, México. Therya 6(3): 535-544.

Graipel, M.E., Cherem, J.J. and Ximienez, A. (2001). Mamíferos terrestres não voadores da Ilha de Santa Catarina, sul do Brasil. Biotemas 14, 109-140.

Guevara-Carrizales, A.A., et al. 2016. Mamíferos terrestres de las ecorregiones áridas del estado de Baja California. In: Briones-Salas, M., et al. (eds.) Riqueza y Conservación de los Mamíferos en México a Nivel Estatal. Instituto de Biología, Universidad Nacional Autónoma de México, Asociación Mexicana de Mastozoología. pp: 63-90.

Hannibal, W, Neves-Godoi, M. 2015. Non-volant mammals of the Maracaju Mountains, southwestern Brazil: composition, richness and conservation. Revista Mexicana de Biodiversidad 86: 217-225

Hannibal, W. 2014. Mammals of medium and large size from a fragmented seasonal forest landscape in Mato Grosso do Sul state, central-western. Check-List 10(6). Doi: http://dx.doi.org/10.15560/10.6.1430.

Harmsen, B.J., et al. 2010. Differential Use of Trails by Forest Mammals and the Implications for Camera-Trap Studies: A Case Study from Belize. Biotropica 42(1): 126–133.

Harvey, C.A., et al. 2006. Dung beetle and terrestrial mammal diversity in forests, indigenous agroforestry systems and plantain monocultures in Talamanca, Costa Rica. Biodiversity and Conservation 15: 555–585.

Haugaasen, T., Peres, C.A. 2005. Mammal assemblage structure in Amazonian flooded and unflooded forests. Journal of Tropical Ecology 21:133–145.

Hendges, C.D., Salvador, C.H. and Nichele, M.A. (2015). Mamíferos de médio e grande porte de remanescentes de Floresta Estacional Decidual no Parque Estadual Fritz Plaumann e em áreas adjacentes, Sul do Brasil. Biotemas 28, 121-134.

Hernández, J.C.H., et al. 2018. Diversidad y patrones de actividad de mamíferos medianos y grandes en la Reserva de la Biosfera La Encrucijada, Chiapas, México. Rev. Biol. Trop. 66(2): 634-646.

Hernández-Pérez, E., et al. 2015. Camera-trap survey of medium and large mammals associated with petenes ecosystem of northwestern coast of the Yucatan Peninsula, Mexico. Therya 6(3): 559-574.

Hetakeyama, R. 2015. Ocupação e padrões de atividades de mamíferos de médio e grande porte em um mosaico de Mata Atlântica e plantações de eucalipto. Master thesis, Universidade Federal de Minas Gerais.

Hidalgo-Mihart, M.G., et al. 2017. Inventory of medium-sized and large mammals in the wetlands of Laguna de Terminos and Pantanos de Centla, Mexico. Check List 13 (6): 711–726.

Huarcaya, R.P., et al. 2019. Camera trapping reveals a diverse and unique high-elevation mammal community under threat. Oryx, in press.

Huck, M. et al. 2017. Mammals and their activity patterns in a forest area in the Humid Chaco, northern Argentina. Check List 13 (4): 363–378.

Hülle, N.L. 2006. Mamíferos de médio e grande porte num remanescente de Cerrado o sudeste do Brasil. Master thesis, Universidade de São Paulo.

Hurtado, C.M., Pacheco, V. 2015. New mammalian records in the Parque Nacional Cerros de Amotape, northwestern Peru. Revista Peruana de Biología 22(1): 077–086.

Janzen, D.H. (ed.). 1983. Costa Rican Natural History. The University of Chicago Press. 816 p.

Jayat, J.P., Ortiz, P.E. 2009. Mamíferos Del Pedemonte De Yungas De La Alta Cuenca Del Río Bermejo En Argentina: Una Línea De Base De Diversidad. Mastozoología Neotropical 17(1):69-86.

Jiménez, C.F. et al. 2010. Camera trap survey of medium and large mammals in a montane rainforest of northern Peru. Rev. Peru. Biol. 17(2): 191–196.

Jiménez, G., et al. 2017. Monitoring program for mammals in a protected area of Colombia. Univ. Sci. 22 (1): 9-29.

Johnson, W.E., Franklin, W.L., Iriarte, J.A. 1990. The mammalian fauna of the northern Chilean Patagonia: a biogeographical dilemma. Mammalia 54(3): 457-469.

Juarez, K.M. Mamíferos de médio e grande porte nas unidades de conservação do Distrito Federal. PhD Thesis, Universidade de Brasilia.

Junges, S.O. and Cademartori, C.V. (2012). Composição da mastofauna de médio e grande porte em um remanescente de floresta com araucária no sul do Brasil. Mouseion 13, 170-181.

Juraszek, A., Bazilio, S. and Golec, C. (2014). Levantamento de mamíferos de médio e grande porte na RPPN Federal Corredor do Iguaçu na região centro-oeste do Paraná. Acta Iguazu 3, 113-123.

Kasper, C.B. et al. 2012. Abundance of Conepatus chinga (Carnivora, Mephitidae) and other medium-sized mammals in grasslands of southern Brazil. Iheringia Série Zoologia 102(3): 303-310.

Kasper, C.B., Feldens, M.J., Mazim, F.D., Schneider, A., Cademartori, C.V. and Grillo, H.C.Z. (2007a). Mamíferos do Vale do Taquari, região central do Rio Grande do Sul. Biociências (On-line) 15, 53-62.

Kasper, C.B., Mazim, F.D., Soares, J.B.G., Oliveira, T.G. and Fabián M.E. (2007b). Composição e abundância relativa dos mamíferos de médio e grande porte no Parque Estadual do Turvo, Rio Grande do Sul, Brasil. Rev. Brasil. Zool. 24, 1087-1100.

Kosydar, A.J., et al. 2014. Effects of hunting and fragmentation on terrestrial mammals in the Chiquitano forests of Bolivia. Tropical Conservation Science 7 (2): 288-307.

Lage, A.R.B. 2011. Efeitos Da Fragmentação De Habitat Sobre A Comunidade De Mamíferos Do Médio Araguaia: Aspectos Teóricos, Descritivos E Conservacionistas. PhD thesis, Universidade Federal de Goiania.

Lasso, C.A., et al. (eds.). 2016. Morichales, cananguchales y otros palmares inundables de Suramérica. Parte II: Colombia, Venezuela, Brasil, Perú, Bolivia, Paraguay, Uruguay y Argentina. Serie Editorial Recursos Hidrobiológicos y Pesqueros Continentales de Colombia. Instituto de Investigación de Recursos Biológicos Alexander von Humboldt (IAvH).

Latorre, L.F.L., López-Arévalo, H.F. 2015. Comunidad de Mamíferos no Voladores en un Área Periurbana Andina, Cundinamarca, Colombia. Acta Biol. Colomb., 20(2):193-202.

Laurindo, R.S., et al. 2017. Mammals in forest remnants of an ecotonal Atlantic Forest-Cerrado area from southeastern Brazil. Neotropical Biology and Conservation 12(1):19-29.

Lavariega, M.C., et al. 2012. Mamíferos Medianos y Grandes de la Sierra de Villa Alta, Oaxaca, México. Mastozoología Neotropical 19(2): 225-241.

Lázari, P.P. 2011. Uso de habitats por mamíferos não-voadores no pantanal de Cácares, Mato Grosso, Brasil. Master thesis, Universidade do Estado de Mato Grosso.

Lázari, P.R., et al. 2013. Flood-mediated use of habitat by large and midsized mammals in the Brazilian Pantanal. Biot. Neotrop. 13(2). http://www.biotaneotropica.org.br/v13n2/en/abstract?article+bn02713022013 ISSN 1676-0603.

Leal, T.L.F. 2015. Comparación de presencia de mamíferos silvestres medianos y grandes en dos sistemas de pastoreo diferentes. Master thesis, Universidad Veracruzana.

Leigh-Jr., E.G. Tropical Forest Ecology. A view from Barro Colorado Island

Leite, J.L.F., et al. 2016. Composition of medium and large mammals in forest reserve in the Cerrado of Brazil central. Revista Árvore 40(5): 825-832.

Léon, E.A.V. 2016. Diversidad y abundancia de macro y meso mamíferos (Clase: Mammalia) en dos zonas con distintos grados de perturbación en el Humedal Ramsar Abras de Mantequilla (Los Ríos - Ecuador) durante Marzo a Diciembre del 2016. Undergraduate thesis, Universidad de Guayaquil.

Lessa, L.G., Alves, H., Geise, L. 2012. Mammals of medium and large size in a fragmented Cerrado landscape in northeastern Minas Gerais state, Brazil. Check List 8(2): 192-196.

Leuchtenberger, C. et al. 2018. Activity pattern of medium and large sized mammals and density estimates of Cuniculus paca (Rodentia: Cuniculidae) in the Brazilian Pampa. Brazilian Journal of Biology. Doi: 10.1590/1519-6984.174403.

Lew, D., et al. 2009. Mamíferos de la Cuenca Alta del río Cuyuní, Estado Bolívar. Conservation International. Doi: 10.1896/054.055.0114.

Lima, F., et al. 2017. ATLANTIC-CAMTRAPS: a dataset of medium and large terrestrial mammal communities in the Atlantic Forest of South America. Ecology 98(11): 2979.

Lima, J.C.S. and Pasciani, V. (2014). Riqueza de espécies de mamíferos de médio e grande porte na Fundação Jardim Botânico de Poços de Caldas, Minas Gerais, Brasil. Rev. Biociências. 20, 62-70.

Lira-Torres, I., Briones-Salas, M. 2012. Abundancia Relativa y Patrones de Actividad de los Mamíferos de los Chimalapas, Oaxaca, México. Acta Zool. Mex. 28(3): 115-122.

Lizcano, D.J., et al. 2016. Riqueza de mamíferos medianos y grandes del refugio de vida silvestre marina y costera Pacoche, Ecuador. Therya 7(1): 135-145.

Luna, R.B., et al. 2017. Terrestrial mammal assemblages in protected and human impacted areas in Northern Brazilian Amazonia. Nature Conservation 22: 147–167.

Machado, F.S., Almeida, A.F., Barros, D.A., Pereira, J.A.A., Silva, R.S., Pereira, A.A.S. 2016. Diversity of medium-sized and large mammals from Atlantic Forest remnants in southern Minas Gerais state, Brazil. Check List 12(5): 1962. DOI: 10.15560/12.5.1962.

Maciel, L. and Maciel, K.P.W.A. (2015). Levantamento preliminar de mamíferos silvestres em uma área de Floresta Ombrófila Mista na região de Porto Vitória-PR. Rev. Elet. Biol. 8, 13-28.

Magioli, M., Ferraz, K.M.P.M.B. and Rodrigues, M.G. (2014). Medium and large-sized mammals of an isolated Atlantic Forest remnant, southeast São Paulo State, Brazil. Check List 10, 850-856.

Mantilla-Meluk, H. 2017. Mamíferos Del Sector Norte Del Parque Nacional Natural Serranía De Chiribiquete. Revista Colombia Amazónica 10: 99-134.

Marafon, A.T., et al. 2018. Mastofauna não voadora de médio e grande porte em área de mosaico de vegetação nativa e exótica na mesorregião Oeste catarinense, Brasil. Saúde, Meio Ambiente e Sustentabilidade 13(2): 54-67.

Marín, N.S.S. 2017. Variaciones en la estructura y la composición de mamíferos terrestres medianos y grandes como resultado de un proceso de restauración ecológica del bosque seco tropical en San Juan Nepomuceno, Bolívar. Undergraduate thesis, Pontificia Universidad Javeriana.

Marinho, P.H., et al. 2018. Mamíferos de Médio e Grande Porte da Caatinga do Rio Grande do Norte, Nordeste do Brasil. Mastozoología Neotropical 25(2): 345-362.

Marques, R.V., Cademartori, C.V. and Pacheco, S.M. (2011). Mastofauna no Planalto das Araucárias, Rio Grande do Sul, Brasil. Rev. Bras. Biociênc. 9, 278-288.

Marques, R.V., Fábina, M.E. 2018. Daily activity patterns of medium and large neotropical mammals during different seasons in an area of high altitude Atlantic rain forest in the South of Brazil. Revista Brasileira de Zoociências 19(3): 38-64.

Martínez, D.L. 2016. Terrestrial mammal communities in Tropical rainforests of Ecuador. Master thesis, University of Manchester.

Martínez, O, et al. 2009. Fauna vertebrada de San Cristóbal en el altiplano sur de Bolivia. Kempffiana 5(1): 28-55.

Martínez, O. et al. 2008. Mamíferos Medianos y Grandes de la Serranía del Aguaragüe, Tarija (Bolivia). Mastozoología Neotropical 15(2): 335-348.

Martins, A.T.O., et al. 2016. Large and medium-sized mammals in the urban park Cinturão Verde, Cianorte, northwestern Paraná Check List 12(2): 1851.

Martins, M.B. 2009. Distribuição e Abundância da Fauna na Área de Vida da Comunidade de Pini, em Lugares Selecionados por Caçadores da Floresta Nacional do Tapajós/Pará. Master thesis, Universidade Federal do Amazonas.

Martins, T.O., Bunhuolo, S.P., Ortêncio, H., Lacher, T.E. 2016. Large and medium-sized mammals in the urban park Cinturão Verde, Cianorte, northwestern Paraná. Check List 12(2): 1851. DOI: 10.15560/12.2.1851.

Mata, C.L.G. 2012. Abundancia relativa de mamíferos terrestres grandes y medianos en el área reflorestada de la Sierra de Zapalinamé, Coahuila, Mexico. Undergraduate thesis, Universidad Autonoma Agraria.

Mayor, P., et al. 2015. Effects of selective logging on large mammal populations in a remote indigenous territory in the northern Peruvian Amazon. Ecology and Society 20(4): 36.

Medeiro, A.Z., et al. 2019. Riqueza de mamíferos de médio e grande porte em Áreas de Preservação Permanente do distrito de Jaci Paraná. RBCA 8(2): 001–008.

Medina, C.E., et al. 2012. Diversidad de Mamíferos en Los Bosques Montanos Del Valle De Kcosñipata, Cusco, Perú. Mastozoología Neotropical 19(1): 85-104.

Medina, C.E., et al. 2016. Mammalian diversity in the Savanna from Peru, with three new addictions from country. Papéis Avulsos de Zoologia 56(2) 9-26.

Medina, W., et al. 2015. Aves y mamíferos de bosque altoandino-páramo en el páramo de Rabanal (Boyacá-Colombia). Revista Ciencia en Desarrollo 6(2): 185-198.

Medina-Fitoria, et al. 2018. Diversidad Biológica De La Cuenca Baja Del Río Grande De Matagalpa En El Caribe De Nicaragua. Revista Nicaraguense De Biodiversidad 38: 145-160.

Melo, C.C.S. 2013. Mamíferos não Voadores da Região dos Lagos, Municipios de Tartarugalzinho, Pracuúba no Amapá. In: Melo, C.C.S. 2013. Região dos Lagos e Sucuriju. PROBIO. pp.: 196-217

Melo, E.R.A., et al. 2015. Diversity, abundance and the impact of hunting on large mammals in two contrasting forest sites in northern amazon. Wildlife Biology 21(5): 234-245.

Melo, R.S., et al. 2014. The role of mammals in local communities living in conservation areas in the Northeast of Brazil: an ethnozoological approach. Tropical Conservation Science Vol.7 (3): 423-439.

Mendes, C.L.S., Santos, B.O., Laia, W.P. and Souza, L.A. (2014). Diversidade de mamíferos de médio e grande porte da reserva particular do patrimônio natural da Mata do Sossego e seu entorno, Minas Gerais. Rev. Bras. Zoociênc. 16, 27-41.

Mendes-Pontes, A.R., Beltrão A.C.M, Normande I.C., Malta A.J.R., Silva Júnior, A.P., Santos, A.M.M. 2016. Mass Extinction and the Disappearance of Unknown Mammal Species: Scenario and Perspectives of a Biodiversity Hotspot’s Hotspot. PLoS ONE 11(5): e0150887. DOI:10.1371/journal.pone.0150887.

Mendes-Pontes, A.R., et al. 2010. Mamíferos de médio e grande porte de Roraima, extremo norte da Amazônia brasileira. In: Barbosa, R.I., Melo, V.F. 2010. Roraima: Homem, Ambiente e Ecologia, INPA/UFRR. Pp: 603-629.

Mendoza, E.I.C. 2017. Composición y diversidad de mamíferos medianos y grandes en el Parque Nacional Montecristo, Santa Ana, El Salvador. Undergraduate thesis, Universidad de El Salvador.

Mesquita, G.P., et al. 2018. Patterns of Mammal Subsistence Hunting in Eastern Amazon, Brazil. Wildlife Society Bulletin 42(2): 272–283.

Michalski, F., Peres, C.A., 2007. Disturbance-Mediated Mammal Persistence and Abundance-Area Relationships in Amazonian Forest Fragments. Conservation Biology 21(6). Doi: 10.1111/j.1523-1739.2007.00797.x

Miranda, J.M.D., Rios, R.F.M. and Passos, F.C. (2008). Contribuição ao conhecimento dos mamíferos dos Campos de Palmas, Paraná, Brasil. Biotemas 21, 97-103.

Modesto, T.C., Pessôa, F.S., Enrici, M.C., Attias, N., Jordão-Nogueira, T., Costa, L.M., Albuquerque, H.C. and Bergallo, H.G. (2008). Mamíferos do Parque Estadual do Desengano, Rio de Janeiro, Brasil. Biota Neotrop. 8, 153-159.

Móran, L., et al. 2018. Interannual and daily activity patterns of mid-sized mammals in Maracaibo Lake Basin, Venezuela, Therya 9(3): 227-236.

Morcatty, T.Q., El Bizri, H.R., Carneiro, H.C.S., Biasizzo, R.L., Alméri, C.R.O., Silva, E. S., Rodrigues, F.H.G., and Figueira, J.E.C. (2013). Habitat loss and mammalian extinction patterns: are the reserves in the Quadrilátero Ferrífero, southeastern Brazil, effective in conserving mammals?. Ecol. Res. 28, 935-947.

Mosquera-Guerra, et al. 2018. Diversidad, abundancia relativa y patrones de actividad de los mamíferos medianos y grandes, asociados a los bosques riparios del río Bita, Vichada, Colombia Biota Colombiana 19(1), in press.

Muzzachiodi, N. 2007. Lista comentada de Mamíferos de la provincia de Entre Ríos. Buenos Aires: Fundación Félix de Azara, Vázquez Mazzini Editores.

Nadkarni, N.M., Wheelwright, N.T. (eds.) 2014. Monteverde: Ecology and Conservation of a Tropical Cloud Forest - 2014 Updated Chapters. Bowdoin Scholars' Bookshelf. Book 4.

Navarro, J.P., Gómez, A. 2015. Diversidad de mamíferos terrestres en bosques cercanos a cultivos de piña, Cutris de San Carlos, Costa Rica. Cuadernos de Investigación 7(1): 59-65.

Negrão, M.D.F.F. and Valladares-Pádua, C. (2006). Registros de mamíferos de maior porte na Reserva Florestal do Morro Grande, São Paulo. Biota Neotrop. 6, 1-13.

Neto, E.H. 2015. Caracterização de mamiferos de médo e grande porte em remanescentes florestais do Cerrado no município de Rio Verde-GO. Undergradute thesis, Universidade de Rio Verde.

Nogueira, E.A., et al. 2014. Mamíferos medianos y grandes en el Piedemonte Andes-Amazonía de Monopamba-Puerres, Colombia. Brenesia 81-82: 111-114.

Nogueira-Junior, W.R. 2018. Padrão de Atividade de Mamíferos Terrestres De Médio E Grande Porte Da Reserva Biológica Do Tapirapé Undergraduate Thesis, Universidade Federal Do Sul E Sudeste Do Pará.

Norris, D., Ramírez, J.M., Zacchi, C. and Galetti, M. (2012). A Survey of mid and large bodied mammals in Núcleo Caraguatatuba, Serra do Mar State Park, Brazil. Biota Neotrop. 12, 127-133.

Nunes, A.V., Scoss, L.M., Prado, M.R. and Lessa, G.M. (2013). Survey of large and medium-sized terrestrial mammals in the Serra do Brigadeiro State Park, Minas Gerais, Brazil. Check List 9, 240-245.

Núñez, M.C., Jiménez, E.C. 2017. Estado poblacional de mamíferos terrestres en dos áreas protegidas de la región central occidental de Costa Rica. Rev. Biol. Trop 65(2): 493-503.

Núñez-Regueiro, M.M. et al. 2015. Spatial patterns of mammal occurrence in forest strips surrounded by agricultural crops of the Chaco region, Argentina. Biological Conservation 187: 19–26.

Ochoa, J, Bevilacqua, M., García, F. 2005. Evaluación ecológica rápida de las comunidades de mamíferos en cinco localidades del Delta del Orinoco, Venezuela. Interciencia 30(8): 466-475.

Odeli, R., et al. 2018. Composição de mamíferos de médio e grande porte da região do cânion do Guartelá, Escarpa Devoniana, Paraná. Boletim da Sociedade Brasileira de Mastozoologia 83: 146-151.

Ojasti, J., et al. 1992. Mamiferos de la expedicion de Tapirapecó, Estado Amazonas, Venezuela. Acta Biol. Venez. 14(1): 27-40.

Oliveira, A.T.M., et al. 2019. Mamíferos de médio e grande porte: indicadores de zonas de amortecimento em reflorestamentos. Sci. Elec. Arch. 2(5), in press.

Oliveira, L.P., Aguiar, D., Margarido, T.C.C. and Pachaly, J.R. (2013). Caracterização faunística de mamíferos de médio e grande porte de um fragmento florestal do noroeste do estado do Paraná, Brasil. Arq. Ciênc. Vet. Zool. UNIPAR 15, 109-114.

Oliveira, L.P., et al. 2012. Caracterização faunística de mamíferos de médio e grande porte de um fragmento florestal do noroeste do estado do Paraná, Brasil. Arq. Ciênc. Vet. Zool. 15(2): 109-114.

Oliveira, M.L., et al. 2008. Biodiversidade – Amazônia. Programa de Pesquisa em Biodiversidade, Reserva Florestal Adolpho Ducke. Áttema Design Editorial.

Oliveira, S.S. 2009. Mamíferos de médio e grande porte de um remanescente de floresta ombrofila mista do município de Riozinho, Rio Grande do Sul. Undergraduate thesis, Centro Universitário La Salle.

Oliveira, T.G., et al. 2016. Nonvolant Mammal Megadiversity and Conservation Issues in a Threatened Central Amazonian Hotspot in Brazil. Tropical Conservation Science 2016: 1–16.

Oliveira, V.B., Linares, A.M., Castro-Corrêa, G.L. and Chiarello, A.G. (2013). Inventory of medium and large-sized mammals from Serra do Brigadeiro and Rio Preto State Parks, Minas Gerais, southeastern Brazil. Check List 9, 912-919.

Onghero, O., et al. 2012. Mamíferos em remanescentes florestais de uma fazenda de plantação de Pinus sp., Água Doce, Santa Catarina, Brasil. Unoesc & Ciência 3(1): 57-64.

Orjuela, O.J., Jiménez, G. 2014. Estudio de la abundancia relativa para mamíferos en diferentes tipos de coberturas y carretera, finca hacienda cristales, área cerritos - La Virginia, municipio de Pereira, departamento de Risaralda – Colombia. Universitas Scientiarum 9: 87-96.

Ortiz-Lozada, L. 2017. Absence of Large and Presence of Medium- Sized Mammal Species of Conservation Concern in a Privately Protected Area of Rain Forest in Southeastern. Tropical Conservation Science 10: 1–13.

Ouboter, P.E., et al. 2011. A comparison of terrestrial large-mammal communities between Brownsberg, Raleighvallen and Coesewijne, Suriname. Academic Journal of Suriname 2: 176–181.

Ouboter, P.E., Kadose, V.S. 2015. Three years of continuous monitoring of the large terrestrial mammals of Brownsberg Nature Park, Suriname. Academic Journal of Suriname 7: 643-660.

Paglia, A.P., Perini, F.A., Lopes, M.O. and Palmuti, C.F. (2005). Novo registro de Blarinomys breviceps (Winge, 1888) (Rodentia, Sigmodontinae) no estado de Minas Gerais, Brasil. Lundiana 6, 155-157.

Palacios, J., Naveda-Rodrígueza, A., Zapata-Ríos, G. 2018. Large mammal richness in Llanganates National Park, Ecuador Mammalia 82(4): 309–314.

Paolino, R.M., et al. 2016. Buffer zone use by mammals in a Cerrado protected area. Biota Neotrop. 16(2). Doi: 10.1590/1676-0611-BN-2014-0117.

Pará. 2017. Instituto de Desenvolvimento Florestal e da Biodiversidade Gestão Ambiental e Territorial da Terra Indígena Alto Rio Guamá: diagnóstico etnoambiental e etnozoneamento. Instituto de Desenvolvimento Florestal e da Biodiversidade.

Pardo, L.E, et al. 2018. Terrestrial mammal responses to oil palm dominated landscapes in Colombia. PLoS ONE 13(5): e0197539.

Paredes, O.S.L. 2016. Efeitos das Variáveis Ambientais e Disponibilidade de Frutos na Distribuição Espacial de Vertebrados Terrestres na Amazônia Oriental, Brasil. Master thesis, Universidade Federal do Amapá.

Pasian, C., et al. 2015. Composición de mamíferos medianos y grandes de la Reserva Natural Provincial Rincón de Santa María (Corrientes, Argentina): Comparación con su zona de amortiguamiento y estado de conservación. Mastozoología Neotropical 22(1):187-194.

Passamani, M., Jenilson, D. and Lopes, S.A. (2005). Mamíferos não-voadores em áreas com predomínio de Mata Atlântica da Samarco Mineração SA, município de Anchieta, Espírito Santo. Biotemas 18, 135-149.

Passamani, M., Mendes, S.L. and Chiarello, A.G. (2000). Non-volant mammals of the Estação Biológica de Santa Lúcia and adjacent areas of Santa Teresa, Espírito Santo, Brazil. Bol. Mus. Biol. Mello Leitão 11, 201-214.

Paulucci, J. 2018. El Ensamble de Carnívoros Medianos y Pequeños de la Reserva De Recursos la Fidelidad (Chaco): Estado Poblacional y Su Relación con Variables Ambientales Master Thesis, Universidad Nacional De Córdoba.

Penido, G. and Zanzini, A.C.S. (2012). Checklist of large and medium-sized mammals of the Estação Ecológica Mata do Cedro, an Atlantic forest remnant of central Minas Gerais, Brazil. Check List 8, 712-717.

Penter, C., Fabián, M.E. and Hartz, S.M. (2008). Inventário rápido da fauna de mamíferos do Morro Santana, Porto Alegre, RS. Rev. Bras. Biociênc. 6, 117-125.

Percequillo, A., Santos, K., Campos, B., Santos, R., Toledo, G. and Langguth, A. (2007). Mamíferos dos remanescentes florestais de João Pessoa, Paraíba. Biol. Geral Exp. 7, 17-31.

Pereira, A.A. 2017. Mamíferos de médio e grande porte na APA pandeiros, MG: inventário e estrutura da comunidade. Master thesis, Universidade Federal de Lavras.

Pereira, L.G., Geise, L. 2009. Non-flying mammals of Chapada Diamantina (Bahia, Brazil). Biota Neotrop., 9(3). Doi: 10.1590/S1676-06032009000300019.

Pereira, S.N., Dias, D., Lima, I.P., Maas, A.C.S., Martins, M. A., Bolzan, D.P., França, D.S., Oliveira, M.B., Peracchi, A.L. and Ferreira, M.F. (2013). Mamíferos de um Fragmento Florestal em Volta Redonda, Estado do Rio de Janeiro= Mammals of a forest fragment in Volta Redonda, Rio de Janeiro state. Bioscience J. 29, 1017-1027.

Peres, C.A. 1999. Structure of Nonvolant Mammal Communities in Different Amazonian Forest Types. In: Eisenberg, J.F., Redford, K.H. (eds). Mammals of the Neotropics: The Central Neotropics. University of Chicago. pp-564-581.

Pérez-Irineo, G., Santos-Moreno, A. 2012. Diversidad de mamíferos terrestres de talla grande y media de una selva subcaducifolia del noreste de Oaxaca, México. Revista Mexicana de Biodiversidad 83: 164-169.

Pérez-Solano, L.A., et al. 2018. Mamíferos medianos y grandes asociados al bosque tropical seco del centro de México. Rev. Biol. Trop. 66(3): 1232-1243.

Peters, F.B., et al. 2011. Aspectos da caça e perseguição aplicada à mastofauna na área de proteção ambiental do Ibirapuitã, Gio grande do Sul, Brasil. Biodiversidade Pampeana 9(1): 16-19.

Peters, F.B., Roth, P.R.O., Machado, L.F., Coelho, E.L., Jung, D.M.H. and Christoff, A.U. (2010). Assembléia de mamíferos dos agroecossistemas constituintes da bacia hidrográfi ca do rio da Várzea, Rio Grande do Sul. Biotemas 23, 91-107.

Pires, D.P.S. and Cademartori, C.V. (2012). Medium and large sized mammals of a semideciduous forest remnant in southern Brazil. Biota Neotrop. 12, 239-245.

Ponce, M. 2016. Evaluación de la riqueza de espécies y distribución de mamíferos em corredores biológicos de la península Batipa. Utilería y Centro de Copiado.

Porfirio, G., et al. 2014. Medium to large size mammals of southern Serra do Amolar, Mato Grosso do Sul, Brazilian Pantanal. Check List 10(3): 473–482.

Portella, T.P. and Flynn, M.N. (2012). Inventário rápido de mamíferos de médio e grande porte da Área de Proteção Ambiental da Ilha Comprida, SP. RevInter Revista Intertox de Toxicologia, Risco Ambiental e Sociedade 5, 19-37.

Prado, H.M., Murrieta, R.S.S., Adams, C. and Brondizio, E.S. (2014). Local and scientific knowledge for assessing the use of fallows and mature forest by large mammals in SE Brazil: identifying singularities in folkecology. J. Ethnobiol. Ethnomed. 10.

Prado, M.R., Rocha, E.C. and Del Giudice, G.M.L. (2008). Medium and large-sized mammal in a forest fragment of atlantic forest, Minas Gerais, Brazil. Rev. Árvore Arv. 32, 741-749.

Preuss, J.F., Pfeifer, G.B., Toral, J.F., Bressan, S.J. 2016. Levantamento Rápido de Mamíferos Terrestres em Um Remanescente de Mata Atlântica do Sul do Brasil. Unoesc & Ciência 7(1): 89-86.

Prieto-Torres, et al. 2011. Lista Preliminar de Mamiferos no Voladores en Tres Localidades de la Vertiente Suroriental de la Sierra de Perijá, Estado Zulia-Venezuela. Boletín del Centro de Investigaciones Biológicas 45(1): 21-34.

Puechagut, P.B., et al. 2018. Association between Livestock and Native Mammals in a Conservation Priority Area in the Chaco of Argentina. Mastozoología Neotropical 25(2):407-418.

Ramcharan, S. 2017. Report on the medium and large terrestrial mammal species of Peperpot Nature park Findings of twenty one month’s camera trapping. Donor WWF Guianas

Ramírez, H.E., Pérez, W.A. 2007. Mamíferos de un fragmento de bosque de roble en el departamento del CAuca, Colombia. Boletín Científico Museo de Historia Natural 11: 65-79.

Ramirez, L.A.L. 2014. Diversidad y estado actual de mamíferos mayores entre las cuencas de los ríos Tigre y Napo, Amazonía peruana. Master thesis, Escuela de Formación Profesional de Ciencias Biológicas, Perú.

Ramirez, O., et al. 2007. Assemblages of Bird and Mammal Communities in Two Major Ecological Units of the Andean Highland Plateau Of Southern Peru. Ecología Aplicada 6(1-2): XXX-XXX.

Ramírez-Chaves, H.E., Ramírez-Mosquera, J. 2008. Mamíferos presentes en el municipio de Popayán, Cauca-Colombia. Bol. Cient. Mus. Hist. Nat. 12: 65-89.

Ramirez-Mejía, A., Sánchez, F. 2016. Activity patterns and habitat use of mammals in an Andean forest and a Eucalyptus reforestation in Colombia. Hystrix, the Italian Journal of Mammalogy. Doi:10.4404/hystrix-27.2-11319.

Reale, R., Fonseca, R.C.B. and Uieda, W. (2014). Medium and Large-sized Mammals in a Private Reserve of Natural Heritage in the Municipality of Jaú, São Paulo, Brazil. Check List 10, 997-1004.

Restrepo, M.F.G., et al. 2016. Mammal Diversity in an Area with Relicts of Dry Forest in the Mid-Magdalena Valley (Caldas, Colombia) Especial de Biología Bosque Seco 20(2): 147-160.

Reyes, H.O., 2013. Composición, Estructura y Diversidad de Mamíferos Terrestres Grandes y Medianos de 16 Áreas Protegidas en Honduras, Usando Fotocapturas como Evidência de Registro. Mesoamericana 17(2): 15-29.

Reyes, H.O., Elvir, F. 2018. Mamíferos en dos bosques riparios de la sabana de pino en la moskitia hondurenha. Revista Mexicana De Mastozoología 8(2): 22-30.

Rimoldi, P.G., Chimento, N.R. 2018. Diversidad de mamíferos nativos medianos y grandes en la cuenca del río Carcarañá, provincia de Santa Fe (Argentina). Rev. Mus. Argentino Cienc. Nat. 20(2): 333-341.

Ríoz-Uzeda, N. 2001. Presencia de mamíferos terrestres medianos y grandes en el Parque Nacional y Área Natural de Manejo Integrado Cotapata a través del uso de métodos indirectos. Undergraduate thesis, Universidad Católica de Colombia.

Rivas, F., Mujica, G.E., Brassiollo, M. 2010. Corredores biológicos y la conservación de la biodiversidad: El caso del corredor norte en Santiago del Estero.

Rocha, A., et al. 2015. Nonvolant mammals in habitats of the Caatinga scrub and cloud forest enclave at Serra da Guia, state of Sergipe. Revista Brasileira de Zoociências 16: 93-103.

Rocha, E.C., Dalponte, J.C. 2006. Composição e caracterização da fauna de mamíferos de médio e grande porte em uma pequena reserva de Cerrado em Mato Grosso, Brasil. Revista Árvore 30(4): 669-678.

Rocha, E.C., Silva, E. 2009. Composição da mastofauna de médio e grande porte na reserva indígena “Parabubure”, Mato Grosso, Brasil. Revista Árvore 33(3): 451-459.

Rocha, E.C., Silva, J., Silva, P.T., Araújo, M.S., Castro, A.L.S. 2018. Medium and large mammals in a Cerrado fragment in Southeast Goiás, Brazil: inventory and immediate effects of habitat reduction on species richness and composition. Biota Neotropica. 19(3): e20180671.

Rocha, E.C., Soares, K.L. and Pereira, I.M. (2015). Medium-and large-sized mammals in Mata Atlântica State Park, southeastern Goiás, Brazil. Check List 11, 1802.

Rocha-Mendes, F., Mikich, S.B., Bianconi, G.V. and Pedro, W.A. (2005). Mammals of the municipality of Fenix, Parana, Brazil: ethnozoology and conservation. Rev. Bras. Zool. 22, 991-1002.

Rodrigues, D.J., et al. 2011. Descobrindo a Amazônia Meridional: biodiversidade da Fazenda São Nicolau. Ed. Pau e Prosa Comunicação Ltda.

Rodríguez, J.G.V., et al. 2018. Distribución espacial de la riqueza de mamíferos en la cuenca del Tocuyo, Venezuela: perspectivas agroecológicas y de conservación Zootecnia Tropical 35 (1-2): 45-62.

Rodríguez-Macedo, M., et al. 2014. Diversidad de los mamíferos silvestres de Misantla, Veracruz, México. Revista Mexicana de Biodiversidad 85: 262-275.

Rojas, C.R. 2005. Abundancia relativa de mamíferos en dos tipos de cobertura de vegetal en la margen nor-oriental del sanctuario de flora y fauna Otún, Quimbaya, Risaralda. Undergraduate thesis, Pontificia Universidad Javeriana.

Rosa, C.A., Souza, A.C. 2017. Large and medium-sized mammals of Nova Baden State Park, Minas Gerais, Brazil Check List 13(3): 2141. DOI: 10.15560/13.3.2141.

Rosas, G.K., Drumond, P.M. 2009. Mamíferos encontrados em dois castanhais localizados ao sudoeste do Estado do Acre, Brasil. Documentos/Embrapa Acre, ISSN 0104- 9046.

Rossaneis, B.K. (2014). Mamíferos de médio e grande porte em pequenos remanecentes florestais da mata atlântica com influências antropogênicas no norte do Paraná. Semina, 35, 15-24.

Rossi, R.V., et al. 2016. Rapid assessment of nonvolant mammals in seven sites in the northern State of Pará, Brazil: a forgotten part of the Guiana Region. Mammalia. Doi: 10.1515/mammalia-2016-0037.

Ruelas, D., et al. 2016. Diversidad de mamíferos medianos y grandes de la cuenca del río La Novia, Purús. In: Mena, J.L., et al. (eds.) 2016. Diversidad Biológica Del Sudeste De La Amazonía Peruana: Avances En La Investigación. Pp: 111-125.

Sakina, A., et al. 2010. Caracterización de la vegetación y la fauna silvestre con fines ecoturísticos, de seis fincas cafetaleras en la comunidad El Bramadero, Condega, Esteli. Undergraduate thesis, Universidad Nacional Agraria.

Salazar, J.C.G., et al. 2017. Vertebrados Terrestres de la Reserva Natural de la Central Hidroel Éctrica De Caldas (Villa María, Colombia): Estado del Conocimiento. Bol. Cient. Mus. Hist. Nat. 21 (1): 71-89.

Salvador, S., et al. 2011. Large mammal species richness and habitat use in an upper Amazonian forest used for ecotourism. Mammalian Biology 76: 115–123.

Sampaio, R., et al. 2010. Long-term persistence of midsized to large-bodied mammals in Amazonian landscapes under varying contexts of forest cover Biodivers Conserv 19: 2421–2439.

Sánchez González, J., F. Durán Alvarado y G. Vega Araya. 2008. Diversidad de Plantas, Mamíferos y Mariposas en los cerros de La Carpintera, Costa Rica. Informe Interno de Proyecto. Departamento de Historia Natural. Museo Nacional de Costa Rica. Ministerio de Cultura y Juventud. San José Costa Rica. 98 pp.

Sánchez, F. Sánchez-Palomino, P., Cadena, A. 2008. Species Richness and Indices of Abundance of Medium-Sized Mammals in Andean Forest and Reforestations with Andean Alder: A Preliminary Analysis. Caldasia 30(1): 197-208.

Sánchez, F., Cadena, A. 2004. Inventario de Mamíferos en un Bosque de Los Andes Centrales de Colombia. Caldasia 26(1) 2004: 291-309.

Sánchez-Giraldo, C., Daza, J.M. 2017. Non-volant mammals from the protected areas associated to hydroelectric projects on the eastern slope of the northern Cordillera Central, Colombia. Check List 13(2): 2098.

Sánchez-Lalinde, C., et al. 2019. Medium- and large-sized mammals in a protected area of Atlantic Forest in the northeast of Brazil. Oecologia Australis 23(2): 234–245.

Sánchez-Mateo, M.A., et al. 2007. Diversidad de aves y mamíferos en zonas donde anida Rhynchopsitta pachyrhyncha, en el municipio de Madera, Chihuahua, México. Revista Latinoamericana de Recursos Naturales 3 (1): 52-57.

Sanino, G.P., Pozo, N., Heran, T. 2016. Presencia de macro y meso-mamíferos terrestres y semi-acuáticos en la zona costera de Reserva Añihué, Patagonia chilena. Boletín del Museo Nacional de Historia Natural 65: 15-30.

Santos, F. et al. 2019. Prey availability and temporal partitioning modulate felid coexistence in Neotropical forests. PLoS One. 2019; 14(3): e0213671.

Santos, F.S. 2009. A diversidade de mamíferos de médio e grande porte e o potencial desta fauna na regeneração de clareiras artificiais na região do Rio Urucu, Coari, Amazonas. Master thesis, Universidade Federal do Pará.

Santos, F.S., Mendes-Oliveira, A.C. 2012. Diversidade de mamíferos de médio e grande porte da região do rio Urucu, Amazonas, Brasil. Biota Neotrop. 12(3): 283-291.

Santos, K.K., Pacheco, G.S.M., Passamani, M. 2016. Medium-sized and large mammals from Quedas do Rio Bonito Ecological Park, Minas Gerais, Brazil. Check List 12(1): 1830. DOI: 10.15560/12.1.1830.

Santos, T.G., et al. 2008. Mamíferos do campus da Universidade Federal de Santa Maria, Rio Grande do Sul, Brasil. Biota Neotrop. 8(1): 125-131.

Santos-Jr., T.S. 2013. Mamíferos do Cerrado de Mato Grosso, com ênfase no uso do espaço por Cerdocyon thous (CARNIVORA, CANIDAE) e Mazama gouazoubira (ARTYODCTYLA, CERVIDAE). PhD Thesis, Universidade Federal de São Carlos.

Santos-Moreno, A., Velásquez, E.R. 2011. Diversidad de mamíferos de la región de Nizanda, Juchitán, Oaxaca, México. Therya 2(2): 155-168.

Sauthier, D.E., et al. 2017. Mamíferos Terrestres En Islas Del Atlántico Sudoccidental, Patagonia, Argentina. Mastozoología Neotropical 24(1): 251-256.

Schaller, G.B., 1983. Mammals and their biomass on a Brazilian ranch. Arq Zool, 31, pp.1-36.

Schittini, A.E.F.B. 2009. Mamíferos De Médio E Grande Porte No Cerrado Mato-Grossense: Caracterização Geral E Efeitos De Mudanças Na Estrutura Da Paisagem Sobre A Comunidade. Master Thesis, Universidade De Brasília.

Siciliano, S., et al. 2015. Large- and Medium-Sized Land Mammals of Northeast Marajó Island, Lower Amazon, Brazil. Natural Resources 6: 37-47.

Silva, F.P.C., Drumond, P.M. 2009. Lista preliminar das espécies de mamíferos e aves encontrados em uma área sob manejo florestal madeireiro no Estado do Amazonas. Embrapa Acre.

Silva, J. 2018. Efeito da perda de habitat para a mastofauna em um fragmento de Cerrado goiano e novos pontos de ocorrência de Cabassous tatouay (Desmarest, 1804). Master thesis, Instituto Federal Goiano.

Silva, S.S., et al. 2009. Use of mammals in a semi-arid region of Brazil: an approach to the use value and data analysis for conservation. Journal of Ethnobiology and Ethnomedicine 15: 33.

Simonetti, J.A. 1999. Diversity and conservation of terrestrial vertebrates in mediterranean Chile Revista Chilena de Historia Natural 72: 493-500.

Simonetti, J.A., et al. 2013. Providing Habitat for Native Mammals through Understory Enhancement in Forestry Plantations. Conservation Biology 27(5): 1117–1121.

Sobral, M., et al. 2017. Mammal diversity influences the carbon cycle through trophic interactions in the Amazon. Nature Ecology & Evolution 1: 1670–1676.

Sousa, M.A.N. and Gonçalves, M.F. (2004). Mastofauna terrestre de algumas áreas sobre influência da Linha de Transmissão (LT) 230 KV PE/PB, CIRCUITO 3. Revista Rev. de Biologia Bio.e Ciências Ciên.da Terra Ter. 4, 1-14.

Souza, Y. et al. 2019. ATLANTIC MAMMALS - a data set of assemblages of medium and large-sized mammals of the Atlantic Forest of South America. Ecology in press.

Spezia, M.B., Grasel, D. and Miranda, G. (2013). Inventário rápido de mamíferos não voadores em um fragmento florestal do bioma Mata Atlântica. Unoesc and Ciência-. ACBS 4, 145-154

Springer, M.T., et al. 2015. Relative Abundance of Mammalian Species in a Central Panamanian Rainforest. Revista Latinoamericana de Conservación 2(2): 19-26.

Srbek-Araujo, A.C., Kierulff, M.C.M. Mamíferos de Médio e Grande Porte das Florestas de Tabuleiro do Norte do Espírito Santo: Grupos Funcionais e Principais Ameaças. In: Rolim, S.G.; Menezes, L.F.T., Srbek-Araujo, A.C. (eds.). Floresta Atlântica de Tabuleiro: diversidade e endemismos na Reserva Natural Vale. 496p

Srbek-Araujo, AC., et al. 2016. Mamíferos De Médio E Grande Porte Das Florestas De Tabuleiro Do Norte Do Espírito Santo: Grupos Funcionais E Principais Ameaças. In: Menezes, R.S.G., et al. (eds.) 2016. Floresta Atlântica de Tabuleiro: Diversidade e Endemismos na Reserva Natural Vale. Editora Rona.

Stallings, J.R., Fonseca, G.A.B., Pinto, L.P.S., Aguiar, L.M.S. and Sábato, E.L. (1991). Mamíferos do Parque Florestal Estadual do Rio Doce, Minas Gerais, Brasil. Rev. Bras. Zool. 7, 663-77.

Tacuri, V.H., et al. 2017. Mamíferos medianos y grandes del Refugio de Vida Silvestre El Pambilar, Esmeraldas, Ecuador. Refugio de Vida Silvestre El Pambilar. Ministerio del Ambiente.

Tedesco, C.D., Silva, D.M., Zanella, N. 2018. Medium-sized mammals in peri-urban environments in southern Brazil. Acta Scientiarum Biological Sciences 40(1): 37562.

Teribele, R. 2007. Comparações entre taxas de encontro de mamíferos de médio e grande porte em focagens noturnas, em dois períodos sazonais, na Fazenda San Francisco (Pantanal, Miranda – Mato Grosso do Sul). PhD Thesis, Universidade Federal do Mato Grosso do Sul.

Thoisy, B., et al. 2008. Assessment of large-vertebrate species richness and relative abundance in Neotropical forest using line-transect censuses: what is the minimal eVort required? Biodivers Conserv (2008) 17:2627–2644

Timm, R.M., et al. 1989. Mammals of the La Selva-Braulio Carillo Complex, Costa Rica. North America Fauna 75: 1-153.

Tinoco-Sotomayor, A.N. 2018. Riqueza, uso y amenazas de mamíferos medianos y grandes en el Distrito de Cartagena de Indias, Colombia. Undergraduate thesis, Universidad de Cartagena.

Torres, I.L., et al. 2005. Mastofauna Del Cerro De La Tuza, Oaxaca. Revista Mexicana de Mastozoología 9:6-20.

Torres-Porras, J., et al. 2017. Large and medium-sized mammals of Buenaventura Reserve, southwestern Ecuador. Check List 13 (4): 35–45.

Tortato, F.R., Testoni, A.F. and Althoff, S.L. (2014). Mastofauna terrestre da Reserva Biológica Estadual do Sassafrás, Doutor Pedrinho, Santa Catarina, Sul do Brasil. Biotemas 27, 123-129.

Trejo, J.R., et al. 2017. Riqueza y Abundancia de Mamíferos Medianos y Grandes en Metzabok, Chiapas, México. In: Alborez, J.O., Méndez, C.F.C. 2017. Lekil Kuxlejal Cultura, Educación y Sustentabilidad. Pp: 85-103.

Trolle M., Kery M. 2005. Camera-trap study of ocelot and other secretive mammals in the northern Pantanal. Mammalia 69 (3-4): 405-412.

Trolle, M. 2003. Mammal survey in the southeastern Pantanal, Brazil. Biodiversity and Conservation 12: 823–836.

Urgilés-Verdugo, C., et al. 2018. Composición y estado de conservación de los Mamíferos medianos y grandes del Corredor Biológico Tropi-Andino, Ecuador. Instituto para la Conservación y Capacitación Ambiental (ICCA).

Utreta, A. Fauna de las Tierras Llaneras. 2003. In: Hetier, J.M., Falcon, R.L. (eds.). Tierras Llaneras de Venezuela. Prrimera Edicion, CIDIAT

Valenzuela, E.P.M., Loachamín, R.C. 2015. Mamíferos de la comunidad shuar Uuntsuants, cordillera del Kutukú, provincia de Morona Santiago, Ecuador. Boletín Técnico Serie Zoológica 10-11: 68-94.

Van der Laan-Barbosa, H.W. 2012. Estrutura de comunidades de mamíferos de médio e grande porte em fragmentos florestais da Amazônia meridional. Master thesis, Universidade Estadual do Mato Grosso.

Veja-López, K.M. 2013. Ensamblaje de Mamíferos Medianos y Grandes en Tres Unidades de Paisaje en el Proyecto La Gloria, Corregimiento de Monterrubio, Municipio de Sabanas de San Ángel, Departamento del Magdalena-Colombia. Master Thesis, Universidad Del Magdalena.

Velilla, M., et al. 2016. Monitoring Threatened Mammals at Three Giants Biological Station, Paraguay Final Report.

Voss, R.S., Lunde, D.P., Simmons, N.B. 2001. The Mammals of Paracou, French Guiana: A Neotropical Lowland Rainforest Fauna Part 2. Nonvolant Species. Bulletin of the American Museum of Natural History 263: 236.

Wallauer, J.P., Becker, M., Martins-Sá, L.G., Liermann, L.M., Perretto, S.H. and Schermack, V. (2000). Levantamento dos mamíferos da Floresta Nacional de Três Barras-Santa Catarina. Biotemas 13, 103-127.

Wolfart, M.R., Da Fré, M., Miranda, G.B. and Lucas, E.M. (2013). Mamíferos terrestres em um remanescente de Mata Atlântica, Paraná, Brasil. Biotemas 26, 111-119.

Xavier, M.S. 2016. Mamíferos terrestres de médio e grande porte do Parque Nacional da Restinga de Jurubatiba: riqueza de espécies e vulnerabilidade local. Master thesis, Universidade Federal do Rio de Janeiro.

Zapata-Ríos, G., et al. 2006. Caracterización De La Comunidad De Mamíferos No Voladores En Las Estribaciones Orientales De La Cordillera Del Kutukú, Amazonía Ecuatoriana. Mastozoología Neotropical 13(2): 227-238.

Zaracho, M., et al. 2017. Mamíferos medianos y grandes del nordeste de la provincia de Corrientes, Argentina. Las Marias. Field Museum.

Zuniga, A. Munoz-Pedreros, A., Fierro, A. 2009. Uso de Habitat de Cuatro Carnivoros Terrestres en el Sur de Chile. Gayana 73(2): 200 - 210, 2009
