# Supplementary figures and images for: Extent, intensity and drivers of mammal defaunation: a continental-scale analysis across the Neotropics
Source: Sci Rep. 2020 Sep 15;10:14750. doi: 10.1038/s41598-020-72010-w (PMC7492218; doi:10.1038/s41598-020-72010-w)

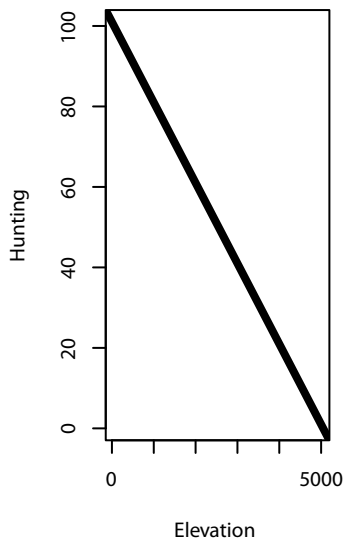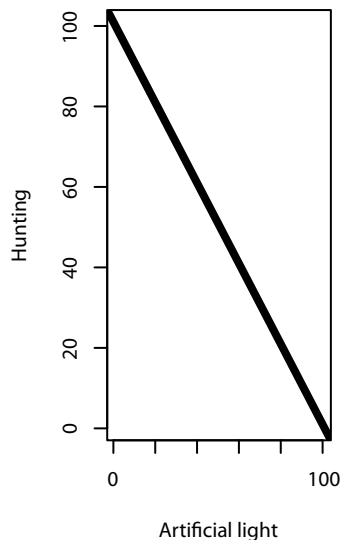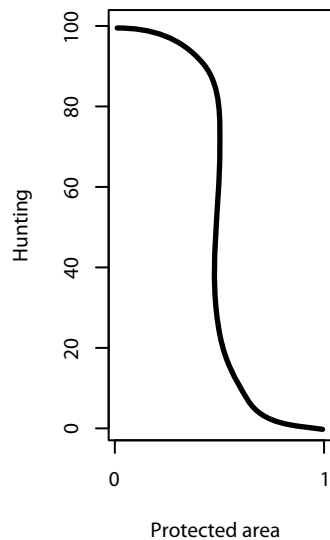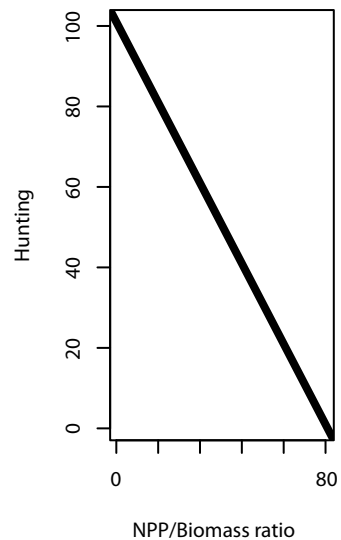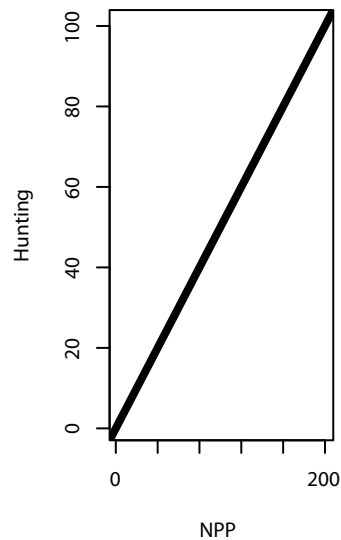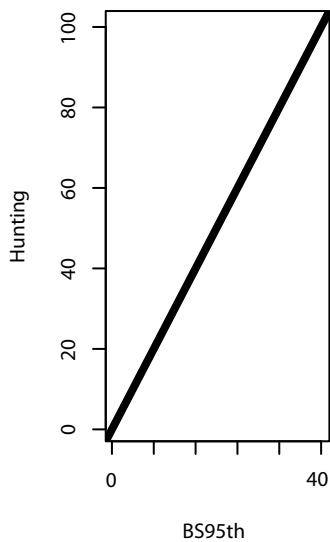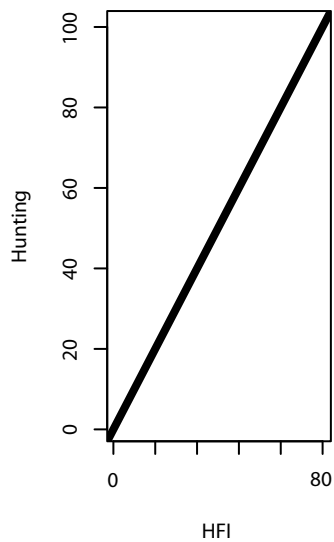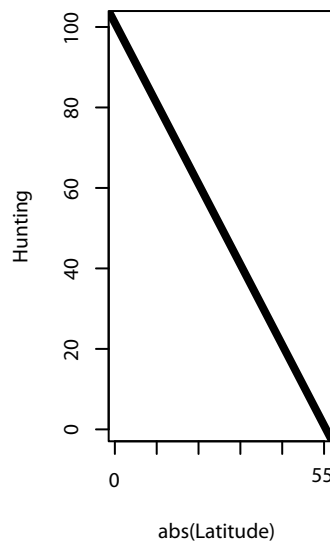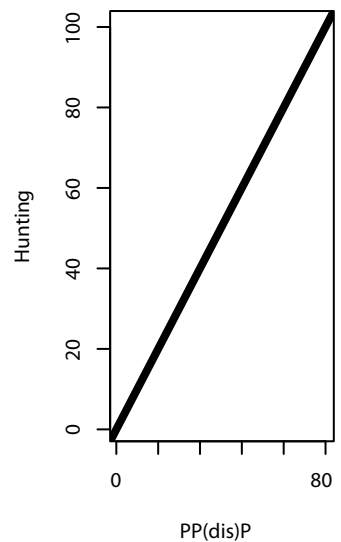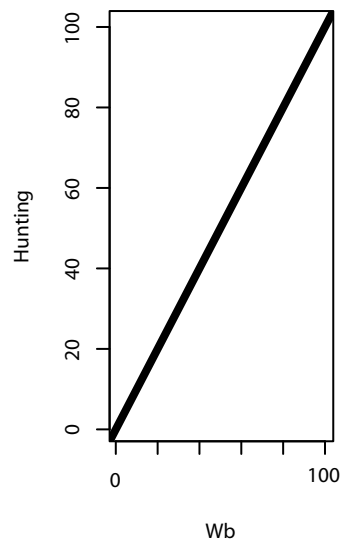

Supplement: Supplementary file 3 — Supporting Information S2. [file 41598_2020_72010_MOESM3_ESM.pdf]
