# Supplementary material for: Extent, intensity and drivers of mammal defaunation: a continental-scale analysis across the Neotropics
Source: Sci Rep. 2020 Sep 15;10:14750. doi: 10.1038/s41598-020-72010-w (PMC7492218; doi:10.1038/s41598-020-72010-w)
Supplement: Supplementary file 4 — Supporting Information S3. [file 41598_2020_72010_MOESM4_ESM.docx]

**Supporting Information S3.** References and links to sources of data layers used to assemble the hunting pressure index (HPI).

1. Elevation (site scale)^1^: available at: <https://asterweb.jpl.nasa.gov/gdem.asp>
2. Artificial lights (4.5-km radial buffer, mean value)^2^: available at: <https://earthobservatory.nasa.gov/features/NightLights/page3.php>;
3. Net primary productivity (4.5-km radial buffer, mean value)^3^: available at: <https://neo.sci.gsfc.nasa.gov/view.php?datasetId=MOD17A2_M_PSN>
4. Vegetation height (4.5-km radial buffer, mean value): Los et al. (2012), available at: <https://data.gov.uk/dataset/ee1719aa-a842-47ee-9da8-803399464ef5/global-vegetation-height-frequency-distributions-from-the-icesat-glas-instrument-produced-as-part-of-the-national-centre-for-earth-observation-nceo>
5. Land cover (ver. 3; 4.5-km radial buffer, mean value): Kobayashi et al. (2017), available at: <https://globalmaps.github.io/glcnmo.html>
6. Human Footprint Index, HFI (ver. 2; 4.5-km radial buffer, mean value): Wildlife Conservation Society (2005), available at: <https://sedac.ciesin.columbia.edu/data/set/wildareas-v2-human-footprint-geographic/data-download>
7. Purchasing Power Parity (PPP; 4.5-km radial buffer, mean value): Nordhaus (2005), available at: <https://sedac.ciesin.columbia.edu/data/set/spatialecon-gecon-v4>

**References**

Los, S. O., Rosette, J. A. B., Kljun, N., North, P. R. J., Chasmer, L., Suarez, J., Hopkinson, C., Hill, R. A., van Gorsel, E., Mahoney, C. and Berni, J. A. J. 2012. Vegetation height and cover fraction between 60° S and 60° N from ICESat GLAS data. – Geoscientific Model Development 5: 413–432.

Nordhaus, W.D. 2005. Geography and macroeconomics: New data and new findings. Proceedings of the National Academy of Sciences of the United States of America (PNAS), 103(10): 3510-3517. http://dx.doi.org/10.1073/pnas.0509842103.

Kobayashi, T., Tateishi, R., Alsaaideh, B., Sharma, R.C., Wakaizumi, T., Miyamoto, D., Bai, X., Long, B.D., Gegentana, G., Maitiniyazi, A. (2017). Production of Global Land Cover Data - GLCNMO2013. Journal of Geography and Geology, Vol. 9, No. 3, 1-15, 2017, <http://dx.doi.org/10.5539/jgg.v9n3p1>.

Wildlife Conservation Society - WCS, and Center for International Earth Science Information Network - CIESIN - Columbia University. 2005. Last of the Wild Project, Version 2, 2005 (LWP-2): Global Human Footprint Dataset (Geographic). Palisades, NY: NASA Socioeconomic Data and Applications Center (SEDAC).

^1^ Compiled by Dave Meyer on behalf of the NASA Land Processes Distributed Active Archive Center and the Joint Japan-US ASTER Science Team.

^2^ The Earth Observatory is part of the EOS Project Science Office at NASA Goddard Space Flight Center.

^3^ These NASA images were made by Reto Stockli, NASA's Earth Observatory Team, using data provided by the MODIS Land Science Team.
