# Supplementary material for: Extent, intensity and drivers of mammal defaunation: a continental-scale analysis across the Neotropics
Source: Sci Rep. 2020 Sep 15;10:14750. doi: 10.1038/s41598-020-72010-w (PMC7492218; doi:10.1038/s41598-020-72010-w)
Supplement: Supplementary file 6 — Supporting Information S5. [file 41598_2020_72010_MOESM6_ESM.docx]

**Supporting Information S5.** Application of Bayes Theorem to the hunting pressure index (HPI).

**Background:** Thomas Bayes idealized the Bayes theorem (Bayes 1764), which depicts the conditional probability of any event. Given any two events A and B, and the conditional probability of A given B, denoted P (A|B), we can use Bayes Theorem to find P (B|A), the conditional probability of B given A, as follows:

$$P\left( A \right|B)= \frac{P\left( B \right|A). P(A)}{P (B)}$$

Or, in the case our approach:

$$P\left( {Eq. 2}_{pot.} \right|{Eq.1}_{am.})= \frac{P\left( {Eq. 1}_{am.} \right|{Eq.2}_{pot.}). P({Eq. 2}_{pot.})}{P (B)}$$

Where:

$P\left( {Eq. 2}_{pot.} \right|{Eq.1}_{am.})$ (i.e., the posterior degree of belief, having accounted for the degree of hunting inhibition (Eq. 1 _am._), is a conditional probability derived from the likelihood of hunting intensification (Eq. 2 _pot._) occurring given that hunting inhibition (Eq. 1 _am._) is true;

$P\left( {Eq. 1}_{am.} \right|{Eq.2}_{pot.})$ is the inverse, i.e. conditional probability derived from the likelihood of hunting inhibition (Eq. 1 _am._) occurring given that hunting intensification (Eq. 2 _pot._) is true.

As a worked example (see also Supporting Information S4):

Site 1:

Eq. 1 _am._ = 0.302

Eq. 2 _pot._ = 0.490

P(B) = ∑ (Eq. 1 _am._ X Eq. 2 _pot._) = 70.51

$P\left( {Eq. 2}_{pot.} \right|{Eq.1}_{am.})= \frac{0.302 X 0.490}{70.51}$ = 0.002103

Site 2:

Eq. 1 _am._ = 0.048

Eq. 2 _pot._ = 0.473

P(B) = ∑ (Eq. 1 _am._ X Eq. 2 _pot._) = 70.51

$P\left( {Eq. 2}_{pot.} \right|{Eq.1}_{am.})= \frac{0.048 X 0.473}{70.51}$ = 0.000329

Therefore, the hunting intensification (P (Eq. 2 _pot._)) is conditioned on the factors that inhibit hunting (P (Eq. 1_am._)). The posterior probability value (P$\left( {Eq. 2}_{pot.} \right|{Eq.1}_{am.}$) derived from Bayes theorem is more strongly related to the factors that intensify, rather than the factors that reduce hunting pressure. The subtraction of the value “intensification-inhibition” (i.e., the hunting pressure index [HPI]) is correlated with the probability value derived from the Bayes theorem, whereby hunting intensification (Eq. 2_pot._) given the factors that inhibit hunting (Eq. 1_am._) are true.


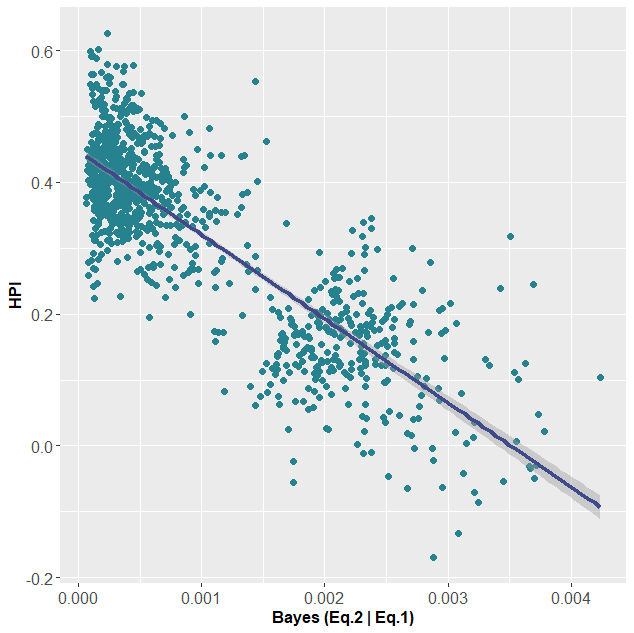


For the statistical analysis we used the R (R Core Team 2020) based on the “BayesTheorem function” (below) of *LaplacesDemon* package (Statisticat 2020).

**R Code of BayesTheorem function:**

function (PrA, PrBA)

{if (missing(PrA))

stop("The PrA argument is required.")

if (missing(PrBA))

stop("The PrBA argument is required.")

if (any(PrA < 0) | any(PrA > 1))

stop("PrA is not in the interval [0,1].")

if (any(PrBA < 0) | any(PrBA > 1))

stop("PrBA is not in the interval [0,1].")

**PrAB <- (PrBA * PrA)/sum(PrBA * PrA)**

class(PrAB) <- "bayestheorem"

return(PrAB)}

**References**

Bayes, T. 1764. "An Essay Toward Solving a Problem in the Doctrine of Chances", Philosophical Transactions of the Royal Society of London 53, 370-418. [Fascimile available online: the original essay with an introduction by his friend Richard Price].

R Core Team. 2020. R: A language and environment for statistical computing. R Foundation for Statistical Computing.

Statisticat, LLC. (2020). LaplacesDemon: Complete Environment for Bayesian Inference. Bayesian-Inference.com. R package version 16.1.4. [https://web.archive.org/web/20150206004624/http://www.bayesian-inference.com/software].
