# Supplementary material for: Extent, intensity and drivers of mammal defaunation: a continental-scale analysis across the Neotropics
Source: Sci Rep. 2020 Sep 15;10:14750. doi: 10.1038/s41598-020-72010-w (PMC7492218; doi:10.1038/s41598-020-72010-w)
Supplement: Supplementary file 8 — Supporting Information S7. [file 41598_2020_72010_MOESM8_ESM.pdf]

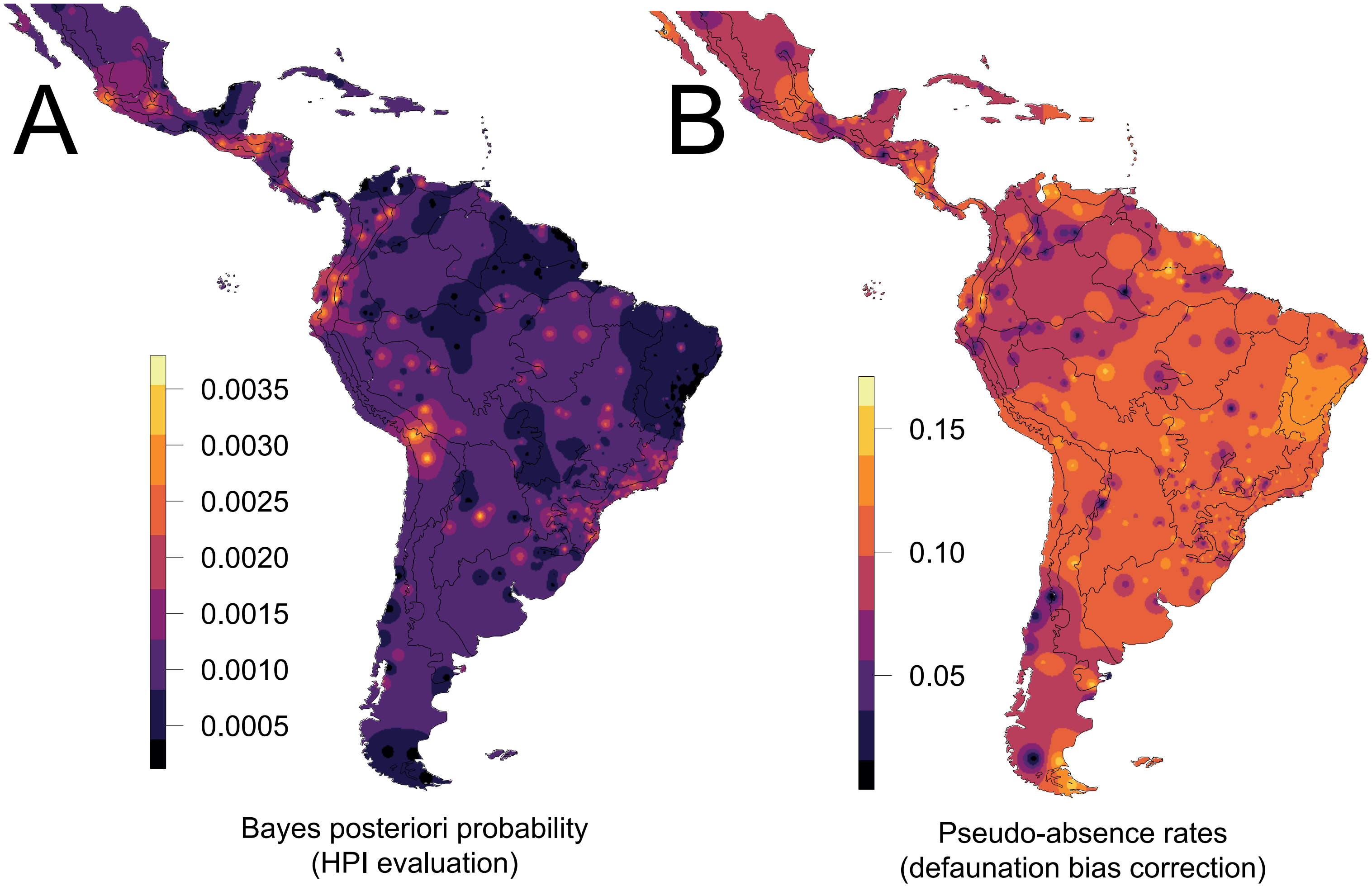
A
0.0035
0.0030
0.0025
0.0020
0.0015
0.0010
0.0005
Bayes posteriori probability
(HPI evaluation)
B
0.15
0.10
0.05
Pseudo-absence rates
(defaunation bias correction)
